# Biomimetic Hydrogels with Oxidative Cross-Linking for Ionically Conductive Interfaces in Long-Term Wearable Bioelectronics

**DOI:** 10.1021/acs.biomac.6c00288

**Published:** 2026-05-08

**Authors:** Kai-Hsiang Chang, Wen-Ya Lee, Jiashing Yu

**Affiliations:** † Department of Chemical Engineering, 34877 National Taiwan University, Taipei 10617, Taiwan; ‡ Department of Chemical Engineering and Biotechnology, National Taipei University of Technology, Taipei 10617, Taiwan

## Abstract

Biomimetic hydrogels with great mechanical properties that provide stable and low-impedance interfaces are essential for long-term wearable bioelectronics. In this study, we developed dopamine-grafted carboxymethyl cellulose (CMCDA) hydrogel and oxidative cross-linking form (CMCDA’). Using multidimensional (1D/2D) NMR techniques, we provide detailed structural elucidation of dopamine-grafted polysaccharides, offering new insights into amide formation, Schiff base/Michael addition structures, and partially oxidized polydopamine segments. The cross-linked CMCDA’ hydrogels are mechanically robust, highly hydrophilic, and strongly adhesive on various substrates, enabling conformal skin contact. After electrolyte exchange with saturated NaCl, CMCDA’ becomes ionically conductive (5–10 S m^–1^) and maintains stable impedance under continuous hydration. Integrated as a wearable electrode interface, CMCDA’ supports reliable electrocardiogram acquisition for one week, outperforming the commercial conductive gel at curved body sites. These results highlight oxidative-cross-linked, cellulose-derived hydrogels as sustainable ionically conductive interfaces for long-term wearable bioelectronics.

## Graphical abstract

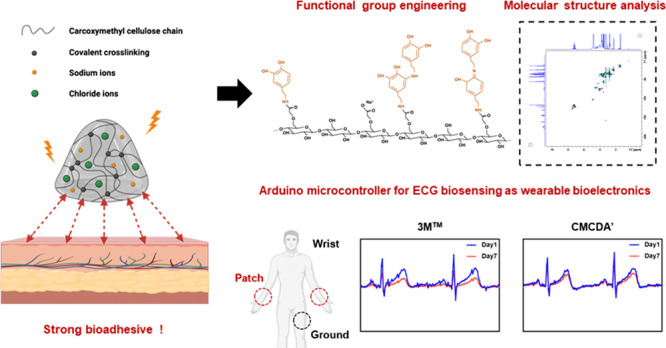

## 1 Introduction

Flexible hydrogel bioelectronic devices have attracted increasing attention in the fields of wearable sensors,^1–5^ electronic skins,^6,7^ human–machine interfaces,^8–10^ and healthcare monitoring.^11–13^ However, hydrogel-based bioelectronics are susceptible to unintended mechanical damage, such as defects, punctures, and fractures arising from complex human motion and dynamic environmental conditions, which can lead to signal deterioration or device failure.^14,15^ To address these challenges, extensive studies have focused on the development of self-healing hydrogel.^16–18^ The mechanical robustness of conductive hydrogels is of critical importance in hydrogel bioelectronic devices, highlighting the necessity and significance of this study and reinforcing the need for a comprehensive investigation of their physical and mechanical properties.

Carboxymethyl cellulose (CMC) is a cellulose derivative with outstanding biocompatibility and environmental sustainability. CMC consists of β-1,4-linked glucose monomer chains and is abundantly available from various biological sources, including plants and microorganisms.^19^ It has been widely used in healthcare and medical applications owing to its biocompatibility, nontoxicity, high water-retention capacity, and cost-effectiveness.^20–22^ Hydrogels derived from CMC closely mimic the microenvironment of biological tissues and the extracellular matrix, thereby promoting cell adhesion and proliferation. Consequently, CMC-based hydrogels have been extensively studied for applications in wound healing, protein/drug delivery, and tissue engineering.^20,23–25^ Several researchers have attempted to develop biomimetic CMC hydrogels. For instance, Xie et al. fabricated catechol-modified carboxymethyl cellulose hydrogels incorporating tannic acid for wound-dressing applications,^26^ while Chen et al. reported the synthesis and characterization of dopamine-grafted CMC hydrogels.^27^ Furthermore, dopamine-modified polymers undergo oxidative cross-linking in the presence of sodium periodate (NaIO_4_).^28,29^ These biomimetic CMC hydrogels refer to the introduction of mussel-inspired catechol chemistry through dopamine functionalization. Marine mussels achieve strong adhesion to various wet surfaces through catechol-rich adhesive proteins such as 3,4-dihydroxy-l-phenylalanine.^27^ By grafting dopamine onto the CMC backbone, similar catechol groups are incorporated into the polymer network, enabling a strong adhesion to diverse substrates and biological tissues. This catechol-mediated interaction mimics the natural adhesive strategy of mussels and provides the hydrogel with bioinspired adhesive properties suitable for wearable bioelectronic interfaces. Nevertheless, the structural complexity of dopamine-modified hydrogels has received limited systematic investigation, and only a few studies have thoroughly examined the influence of functional group engineering and cross-linking on the physicochemical properties of biomimetic hydrogels. Foundational research on this aspect remains scarce, although it is essential to advance the rational design of functionalized hydrogels.

In the context of CMC-based hydrogel bioelectronic devices, Yanping Wang et al. developed a conductive hydrogel composed of CMC and poly­(vinyl alcohol) (PVA) integrated with poly­(3,4-ethylenedioxythiophene):polystyrene sulfonate (PEDOT:PSS),^30^ which served as a conformal and biocompatible interface for electrocardiogram (ECG) biosensing. Wei et al. prepared multifunctional hydrogels comprising CMC, acrylic acid, and alkaline calcium bentonite with stretchable, self-healing, self-adhesive, antibacterial, and 3D-printable characteristics.^31^ However, these formulations typically rely on petroleum-based materials, such as PVA, or chemically irritating monomers, such as acrylic acid. To achieve more sustainable alternatives, Juillard et al. cross-linked CMC using citric acid to create environmentally friendly microneedle patches that swelled in interstitial fluid and functioned as ionically conductive electrodes for electrophysiological recording.^32^ Despite these advances, such hydrogels still lack the combination of strong mechanical integrity and intrinsic bioadhesive properties inspired by natural systems. Commercial conductive gels, typically composed of starch and electrolytes, also exhibit poor bioadhesion, leading to unstable and noisy signals during long-term electrophysiological monitoring. Thus, there is a critical need for eco-friendly, biocompatible, and mechanically robust conductive hydrogels that exhibit strong bioadhesion for next-generation wearable bioelectronics.^33–35^

In this study, a dopamine-modified carboxymethyl cellulose (CMCDA) was synthesized and thoroughly characterized using multidimensional (1D/2D) nuclear magnetic resonance (NMR), Fourier-transform infrared (FTIR), and ultraviolet–visible (UV–vis) spectroscopy to elucidate its molecular structure, addressing the currently limited understanding of the structural elucidation and characterization of biomimetic CMC-based hydrogels following functional group engineering. The mussel-inspired catechol chemistry provides the hydrogel with biomimetic adhesive properties, enabling stable conformal contact with biological surfaces. Oxidative cross-linking was subsequently achieved using NaIO_4_ as an oxidant to form a cross-linked CMCDA’ hydrogel. The effects of functional modification and cross-linking on the rheological behavior, mechanical performance, internal microstructure, gelation characteristics, and thermosensitivity were comprehensively analyzed. These results provide valuable insights into the structure–property relationships of biomimetic CMC-based hydrogels and offer a reference framework for future hydrogel designs. After investigating the physical property changes resulting from functional groups, a NaCl solution was incorporated into the cross-linked CMCDA’ hydrogel to construct an ionically conductive network, enabling the conductive hydrogel to function as a strain-responsive material with tunable resistance. The conductive hydrogel was integrated into an Arduino-based microcontroller to fabricate a wearable biosensing system capable of real-time ECG monitoring. Compared to commercial conductive gels, the biomimetic hydrogel demonstrated superior signal-to-noise ratios (SNR), particularly under joint bending conditions (e.g., at the elbow), and exhibited excellent long-term stability. Importantly, the hydrogels are composed entirely of cellulose-based, eco-friendly materials without petroleum-derived or environmentally hazardous components, providing a sustainable, high-performance alternative for future biosensing and wearable bioelectronic applications (Scheme 1).

**Scheme 1:**
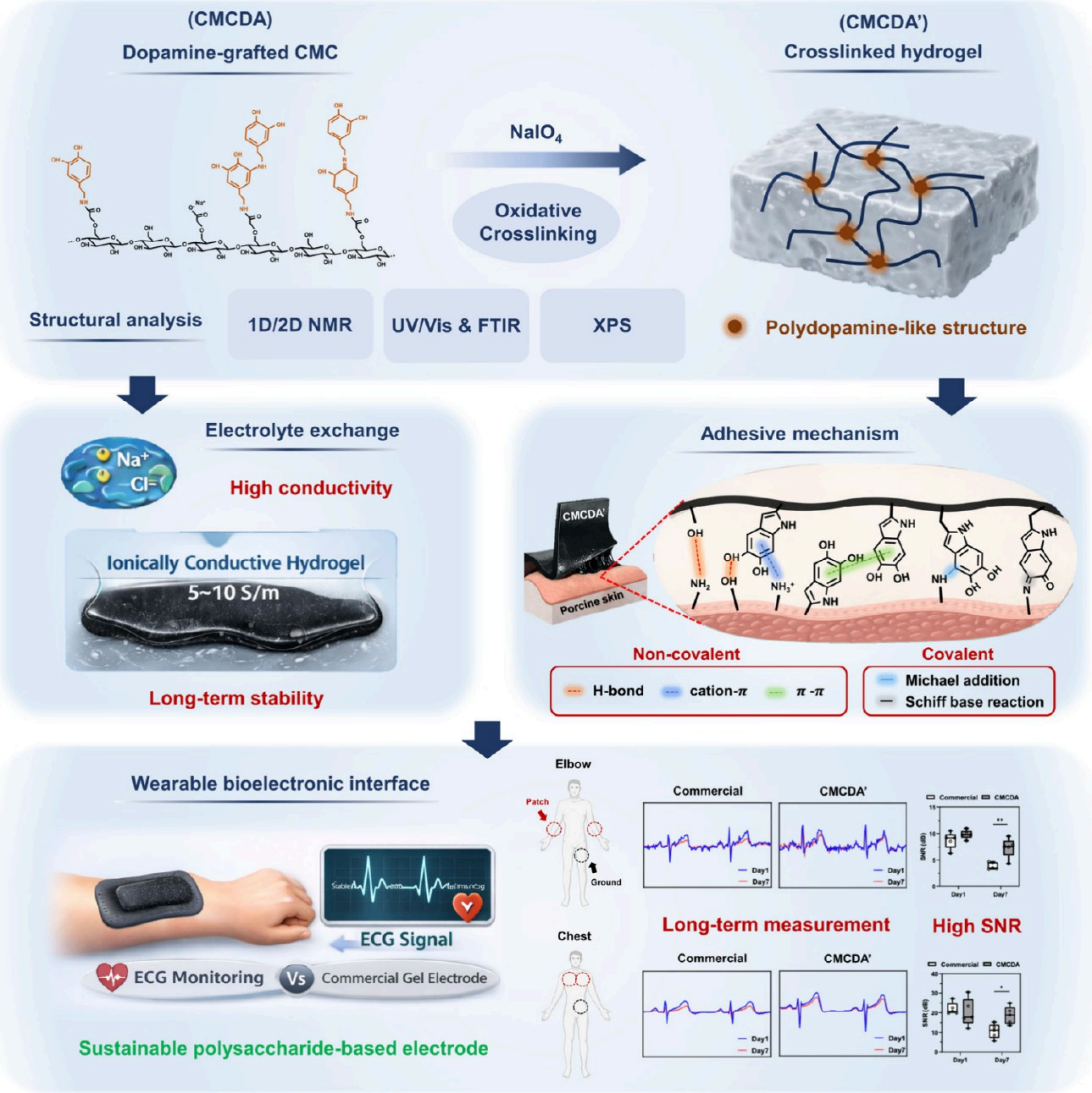
Schematic Illustrations of CMCDA’ Conductive Hydrogel with Bioadhesive Properties Integrated into an Arduino-Based Wearable Microcontroller for Real-Time ECG Monitoring and Structural Analysis

## 2 Materials and Methods

### 2.1 Materials

Carboxymethyl cellulose (CMC) was purchased from Aencore. Dopamine hydrochloride (DA, 99%) and sodium metaperiodate (NaIO_4_) were purchased from Alfa Aesar. 1-Ethyl-3-(3-dimethylaminopropyl) carbodiimide (EDC) was purchased from Matrix Scientific. *N*-Hydroxysuccinimide (NHS), deuterium oxide (D_2_O, 99.75% atom D), sodium chloride (NaCl, 99%), and antibiotic antimycotic solution (penicillin/streptomycin/amphotericin, PSA) were purchased from Sigma-Aldrich. Phosphate buffered saline (PBS) was purchased from Biomate. Potassium bromide (KBr, 99%) was purchased from Honeywell Fluka. Dulbecco’s modified Eagle medium high glucose (DMEM-HG) and fetal bovine serum (FBS) were purchased from Hyclone. Trypsin-EDTA was purchased from Biological Industries. Cell Counting Kit-8 (CCK-8) was purchased from Elabscience. Calcein AM and ethidium homodimer-1 (EthD-1) were purchased from Thermo Fisher Scientific.

### 2.2 Synthesis of CMCDA

Dopamine-functionalized CMCDA was synthesized *via* carbodiimide-mediated coupling using EDC and NHS following a modified literature procedure.^28^ In a typical synthesis, 250 mg of CMC was dissolved in PBS with continuous stirring overnight to obtain a homogeneous solution. Separately, 1.1971 g of EDC and 0.5989 g of NHS were added dropwise into the CMC solution to activate the carboxyl moieties. Subsequently, 1 g of DA was added to the reaction mixture. The coupling reaction was allowed to proceed overnight under a nitrogen atmosphere to suppress DA oxidation and facilitate amide bond formation. The resulting product was dialyzed against deionized (DI) water to remove unreacted reagents and lyophilized to obtain a purified CMCDA powder.

### 2.3 Identification of CMCDA

Thirty milligrams of lyophilized CMCDA powder was dissolved in 600 μL of D_2_O at 333 K for NMR analysis. One-dimensional (^1^H and ^13^C) NMR spectra, distortionless enhancement by polarization transfer (DEPT-90 and DEPT-135) analysis, and two-dimensional spectra, including correlation spectroscopy (COSY), heteronuclear single quantum coherence (HSQC), and heteronuclear multiple bond correlation (HMBC), were recorded by using an 800 MHz FT-NMR spectrometer (Bruker AVIII-800) equipped with a temperature-controlled probe operating at 333 K. The degree of substitution (DS) of carboxymethyl groups on the CMC backbone was determined from the ^13^C NMR spectra as follows.

$${\mathrm{DS}}_{\mathrm{carboxymethyl}}=\frac{I_{176-180}}{I_{100-105}}$$

where *I*_176–180_ and *I*_100–105_ are the integrated areas of the ^13^C NMR signals at 176–180 ppm (carboxyl carbon) and 100–105 ppm (C-1 of the anhydroglucose unit (AGU)), respectively. The DS for DA functionalization was calculated from the ^1^H NMR spectra as follows:

$${\mathrm{DS}}_{\mathrm{DA}}=\frac{I_{7.1-7.3}/3}{I_{3.4-5.1}}$$

where *I*_7.1–7.3_ and *I*_3.4–5.1_ are the integrated areas of the ^1^H NMR signals at 7.1–7.3 ppm (aromatic protons of DA) and 3.4–5.1 ppm (protons of the AGU ring region, including H-1), respectively. The division by 3 accounts for the three aromatic protons of dopamine contributing to the 7.1–7.3 ppm region. The amidation conversion was determined from ^13^C NMR spectra using:

$$\mathrm{amidation conversion}=\frac{I_{172-175}}{I_{172-175}+I_{176-180}}$$

where *I*_172–175_ and *I*_176–180_ correspond to the integrated areas of the ^13^C NMR signals at 172–175 ppm (amide carbonyl) and 176–180 ppm (residual carboxyl carbon), respectively.

The chemical structure and bonding characteristics of the CMCDA hydrogel were further analyzed by using FTIR spectroscopy (PerkinElmer Spectrum 100). The samples were dried and finely ground with KBr at a mass ratio of 1:200, and then compressed into transparent pellets using a hydraulic press. FTIR spectra were collected at a resolution of 4 cm^–1^, averaged over 16 scans, across a wavenumber range of 400–4500 cm^–1^. Subsequently, UV–vis spectroscopy was performed by dissolving lyophilized CMCDA in deionized (DI) water to obtain a 0.5 mg mL^–1^ solution, and absorbance spectra were measured between 200 and 400 nm by using a Cary 300 UV–vis spectrophotometer (Agilent Technologies). Unmodified CMC solutions were used as for baseline reference blanks.

The weight-average molecular weights (*M*_w_) of the samples were determined by gel permeation chromatography (GPC) using a Jasco PU-2080 Plus system equipped with an RI-2031 refractive index detector. Separation was carried out on a Phenomenex Phenogel 5 μm 10^3^ Å column, with DI water as the mobile phase at a flow rate of 1 mL min^–1^. The samples were dissolved in DI water at a concentration of 10 mg mL^–1^ prior to analysis. The calibration curve for number-average molecular weight determination was constructed using ten polystyrene standards with molecular weights ranging from 1580 to 288000 g mol^–1^. The resulting data were processed and analyzed by using Scientific Information Service Corporation (SISC) Chromatography Data Solution software, version 3.1.

### 2.4 Preparation of Oxidative Cross-Linked CMCDA’

For clarity, CMCDA refers to the dopamine-grafted carboxymethyl cellulose precursor prior to oxidative cross-linking, whereas CMCDA’ denotes the oxidatively cross-linked hydrogel obtained after NaIO_4_-induced oxidation of dopamine functional groups. Oxidatively cross-linked CMCDA’ hydrogel was prepared by dissolving lyophilized CMCDA powder in PBS, followed by the addition of NaIO_4_ as the oxidizing agent. The final concentrations of CMCDA and NaIO_4_ were adjusted to 5 wt% and 5 vol%, respectively. The homogeneous solution was then poured into a mold and left at 25 °C for 24 h to allow complete oxidative cross-linking, yielding an oxidatively cross-linked CMCDA’ hydrogel.

To ensure consistency in sample preparation, DI water was used as the solvent for all hydrogel formulations, including CMC, CMCDA, and oxidatively cross-linked CMCDA’, in all experiments related to the evaluation of physical properties. For electrochemical characterization, biosensing assessments, and wearable bioelectronic ECG measurements, DI water was replaced with a saturated NaCl solution at 25 °C to introduce ionic conductivity.

### 2.5 Characterizations of Hydrogels

Rheological analyses were performed by using a rotational rheometer (HR-2, TA Instruments). Temperature ramp tests with varying temperature from 25 to 60 °C were conducted to measure change in storage modulus (G’) with fixed 0.1% strain. Strain sweep tests were performed at a constant strain of 1% and an angular frequency of 10.0 rad s^–1^. The shear-thinning behavior was then examined through flow sweep tests, in which 200 μL of each sample was subjected to shear rates ranging from 1 to 100 s^–1^ at both room temperature (25 °C) and elevated temperature (60 °C) using an 8 mm parallel-plate geometry with a 0° angle. The self-healing properties of the hydrogels were evaluated using continuous step strain tests under low (0.1%) and high (800%) strain conditions at a frequency of 1 rad s^–1^, conducted at both 25 and 60 °C with the same plate geometry.

A number of literatures using the modulus measured by rheometer to quantitatively calculate the polymer cross-linking density for cross-linking hydrogels.^36,37^ First, the molecular weight between cross-links (*M*_*C*_) can be calculated from the *G*′ by using the following equation:

$$M_{C}=\frac{RTd}{G^{\prime }}$$

where *R* is the universal gas constant (8.314 J K^–1^ mol^–1^), *T* is the absolute temperature during measurement (298 K), and *d* is the density of the hydrogel. Then, the cross-linking density of the polymer can be calculated using the following equation:

$$G^{\prime }=vRT$$

where *v* is the cross-linking density (mol cm^–3^).

The compression and tensile properties of the hydrogels were evaluated by using a TA.XT PlusC texture analyzer (Stable Micro Systems) equipped with a 5 N load cell. Prior to testing, a preload of 0.1 N was applied at 0.1 mm s^–1^ to ensure proper contact between the sample and the probe. The compression and tensile tests were conducted at a cross-head speed of 0.1 mm s^–1^. For the compression test, the hydrogel samples were molded into cylindrical specimens, 8 mm in diameter and 4 mm in height, and subjected to unconfined compression between two glass plates. For the tensile tests, the samples were cast into dog-bone-shaped specimens and stretched under identical testing conditions. Cyclic compression and tensile measurements were performed at strain levels of 10%, 20%, and 30%, followed by recovery cycles. The elastic modulus was determined from the linear region (10–20%) of the stress–strain curve for both the compression and tensile tests. The toughness was calculated from the area under the stress–strain curve, whereas the dissipated energy was the area enclosed between the loading and unloading curves.

Differential scanning calorimetry (DSC) was performed using a DSC analyzer (Q25, TA Instruments) to evaluate the thermal behavior of the hydrogels over a temperature range of 10–300 °C at a heating rate of 5 °C/min. Prior to analysis, the samples were ground and lyophilized to remove residual moisture.

The samples were first desiccated to remove residual moisture and then freeze-dried and sputter-coated with a thin platinum layer to enhance the surface conductivity for Scanning electron microscopy (SEM) imaging. Microstructural observations were conducted by using a Nova NanoSEM 230 microscope (Thermo Fisher Scientific) operated at an accelerating voltage of 5 kV under high-vacuum conditions. High-resolution images were obtained by using a through-the-lens detector and an Everhart-Thornley detector.

The swelling of the hydrogels was determined by monitoring the change in weight after immersion in DI water. The lyophilized samples were completely dehydrated to establish an accurate initial dry weight (*W*_0_) and subsequently immersed in 25 mL of DI water at room temperature. The swollen weight (*W*_1_) was recorded after removing excess surface water at predetermined time intervals (5, 15, and 30 min, then 1, 2, 4, 6, 12, 16, and 24 h). The swelling ratio was calculated using the following equation:

$$\mathrm{Swelling ratio}\left(\%\right)=\frac{W_{1}-W_{0}}{W_{0}}\times 100$$

X-ray photoelectron spectroscopy (XPS) analysis was performed using a Nexsa G2 System (Thermo Scientific) to characterize the chemical bonding states after modification and oxidative cross-linking. Before analysis, the samples were ground and lyophilized to remove residual moisture.

The adhesive properties of the hydrogels were evaluated by lap shear and 180° peel tests adapted from ASTM F2255 and F2256, respectively. First, the residual fat on the porcine skin was carefully removed by using a scraper to avoid interference with the adhesion test. Two pieces of porcine skin (5 cm in length and 1 cm in width) were prepared and positioned with their dermal sides facing each other. Then, 50 μL of hydrogel was applied *in situ* between the tissue surfaces. To ensure intimate contact, a 1 kg sample was placed on the assembled samples for 30 min. The bonded tissues were then subjected to mechanical testing using a TA.XT PlusC texture analyzer (Stable Micro Systems) at a crosshead speed of 1 mm s^–1^ until failure. For the lap shear test, the maximum shear stress obtained from the shear stress–displacement curve was used to determine the tissue adhesive strength of the hydrogels. The shear stress was calculated by dividing the maximum shear force by the overlapping contact area between the two tissue samples. For the 180° peel test, the interfacial toughness was determined from the area under the force/width–displacement curve, representing the energy required to separate the two bonded surfaces.

### 2.6 Electrochemical Properties and Biosensing Performance

Following the procedure reported previously,^38^ the electrical impedance behavior of the conductive CMCDA’ hydrogel was analyzed using electrochemical impedance spectroscopy over a frequency range of 0.1 Hz to 100 kHz (Squidstat Prime, Admiral Instruments). Measurements were performed in custom-fabricated poly­(methyl methacrylate) (PMMA) chambers containing rectangular hydrogels 1 cm wide, 0.5 cm high, and 0.5, 1.0, 1.5, 2.0, and 2.5 cm long. The cross-sectional area (*A*_C_) of each hydrogel sample was thus fixed at 0.5 cm^2^. The setup of the custom-made device is shown in Figure S5. Bode plots for CMCDA’ hydrogels of varying lengths were used to determine the hydrogel resistance (*R*_l_) at 100 kHz as a function of their length (*L*). The resulting relationship enabled the extraction of the contact resistance (*R*_C_) between the hydrogel and the electrodes, as well as calculation of the intrinsic electrical conductivity (σ) of each hydrogel according to the following equation:

$$R_{l}=\frac{L}{\sigma A_{C}}+R_{C}$$

CMCDA’ hydrogels were stored at 25 °C in a sealed dish, with moist cotton placed inside the dish to maintain humidity. Under this setup, the conductive hydrogels were kept in an environment of approximately 100% relative humidity, and the samples were taken out once per day to measure conductivity

Nyquist plots and phase-frequency plots for the CMCDA’ hydrogel were obtained using a potentiostat/galvanostat electrochemical analyzer (Autolab PGSTAT204) within a three-electrode configuration over 0.1 Hz to 100 kHz. The hydrogel sample (10 mm × 10 mm × 5 mm) was positioned on one side of an air-plasma-treated carbon graphite felt (40 mm × 10 mm × 5 mm) and treated at 18 W for 3 min using a plasma cleaner (PDC-32 G, Harrick Plasma Corp.). Plasma-treated carbon felt without a hydrogel served as the control electrode. In the three-electrode setup, the hydrogel-carbon felt composite, a platinum wire, and a Ag/AgCl electrode functioned as the working electrode, counter electrode, and reference electrodes, respectively. The measurement setup is shown in Figure S6.

The electrical signal response of the conductive CMCDA’ hydrogel under tensile deformation was evaluated by integrating a portable and wireless electrical measurement device Elite EDC_WTP-EDC-03 (BioPro Scientific) with a TA.XT PlusC texture analyzer (Stable Micro Systems). Dog-bone-shaped hydrogel specimens were mounted on a texture analyzer by using copper adhesive tape, which served as an electrical contact for the potentiostat. During stretching at a speed of 0.1 mm s^–1^, a constant voltage of 0.1 V was applied across both ends of the hydrogel, and the resulting current was continuously recorded. The instantaneous resistance was calculated using Ohm’s law. The relative change in resistance is defined as follows:

$$\mathrm{relative resistance change}\left(\%\right)=\frac{R-R_{0}}{R_{0}}\times 100=\frac{\Delta R}{R_{0}}\times 100$$

where *R*_0_ is the initial resistance and *R* is the resistance under tensile strain.

The GF, representing the strain sensitivity of the hydrogel, was determined from the slope of the relative resistance change–strain (ε) curve, as described in the following equation:

$$\mathrm{GF}=\frac{\Delta R/R_{0}}{\varepsilon }$$

### 2.7 Wearable Bioelectronics for ECG Measurement

A real-time three-channel ECG-monitoring platform was built by integrating an Arduino UNO microcontroller with a SparkFun AD8232 single-lead heart rate monitor (Analog Devices). Commercial 3M^TM^ Red Dot ECG monitoring electrodes were used as the control conductive gel, and the conductive CMCDA’ hydrogel served as a bioinspired replacement for cardiac signal acquisition. We removed the gel from the center of the commercial 3 M gel electrode to create a circular cavity with a diameter of 1 cm and directly filled it with 300 μL of conductive hydrogel with a thickness of 2–3 mm, replacing the original 3 M gel. The Arduino microcontroller was programmed using Arduino Integrated Development Environment (IDE) version 2.3.6, and real-time signal acquisition and data processing were implemented in Python using PyCharm IDE. ECG signals were continuously recorded over seven days from various body locations, including the wrist, elbow, neck, and chest. The SNR was calculated using the following equation, modified from a previous study:^39^

$$\mathrm{SNR}\left(\mathrm{dB}\right)=20\log_{10}\left(\frac{S_{\mathrm{signal}}}{S_{\mathrm{noise}}}\right)$$

where *S*_signal_ represents the filtered ECG signal and *S*_noise_ denotes the difference between the unfiltered and filtered signals.

### 2.8 Cell Cultures and In Vitro Cytocompatibility

Mouse fibroblast cells (L929) were cultured in DMEM/HG medium supplemented with 10% FBS and 1% PSA. Cells were incubated at 37 °C with 5% CO_2_ in a humid incubator. The medium was replenished every 2–3 days until cell confluence reached over 90% in 10 cm culture dishes. Upon reaching this confluence, the medium was aspirated, and the cells were washed with 4–5 mL of PBS. Subsequently, trypsin-EDTA diluted in PBS (0.05%, 2 mL) was added to the dishes, and incubation proceeded for 5 min at 37 °C. The enzyme reaction was halted by adding DMEM-HG medium with FBS and centrifugation for 5 min. Following removal of the supernatant, cells were resuspended in the medium. The number of suspended cells was calculated using a hemocytometer (Suremark Hand Tally Counter SQ-3338, Singapore).

The biocompatibility of the CMCDA’ hydrogels was assessed according to ISO 10993–5 guidelines using both quantitative (CCK-8) and qualitative (live/dead) assays with the L929 cell line. The cells were routinely screened for microbial contamination, including *Mycoplasma*, using polymerase chain reaction and were contamination-free throughout the study. For extract preparation, hydrogels were sterilized by UV exposure overnight and rinsed three times with PBS. Sterilized hydrogels were then immersed in DMEM-HG to obtain the hydrogel extracts. Aliquots (150 μL) were collected after 4 h, 1 day, and 2 days. For the CCK-8 assay, L929 cells (3,000 cells per well) were seeded in 96-well plates and allowed to attach overnight. The culture medium was then replaced with hydrogel extracts, while control wells received fresh DMEM-HG. At each time point, 10 μL of CCK-8 reagent was added to 100 μL of medium, followed by incubation for 2 h at 37 °C. Absorbance was measured at 450 nm by using a microplate reader (SpectraMax i3x, USA), and cell viability was calculated as the percentage relative to the control group. Qualitative cytocompatibility was further examined using live/dead staining after 48 h of extract exposure. The cells were incubated with Calcein AM (1:500 dilution) and EthD-1 (1:2000 dilution) for 20 min, washed with PBS, and imaged under an inverted fluorescence microscope (IX71, Olympus, Japan). Calcein AM marked live cells in green, while EthD-1 stained dead cells in red.

For the direct contact test, the hydrogels were placed directly in 12-well plates in contact with the L929 cells. CCK-8 assay and live/dead staining of cells were performed after 1, 4 and 7 days of exposure to the hydrogels. In addition, the cell morphology was further examined by bright-field microscopy (ECLIPSE Ts2, Nikon, Japan).

### 2.9 Statistical Analysis

Experimental data are presented as the mean ± the standard deviation (SD). All statistical analyses were conducted using Prism version 8.01 software. The statistical significance was evaluated through one-way analysis of variance (ANOVA), followed by posthoc tests to compare group differences. Results with *p* values greater than 0.05 were considered nonsignificant (n.s.).

## 3 Results and Discussion

### 3.1 Synthesis and Identification of CMCDA

CMC, a cellulose derivative, was chosen as the backbone matrix to construct a bioinspired hydrogel. DA, a catecholamine, was grafted onto the CMC *via* amine coupling (Figure 1a). In this reaction, the carboxyl groups on the CMC backbone are first activated by EDC to form an O-acylisourea intermediate, which is then converted to an amine-reactive NHS ester. This intermediate subsequently reacts with the primary amine of DA to form amide linkages. Notably, DA can also undergo Michael addition and Schiff base reactions between the catechol/quinone and amine groups, leading to additional DA-DA coupling. Thus, the proposed DA-functionalized CMCDA structure contains multiple types of DA-derived side chains arising from functional group engineering.

**Figure 1:**
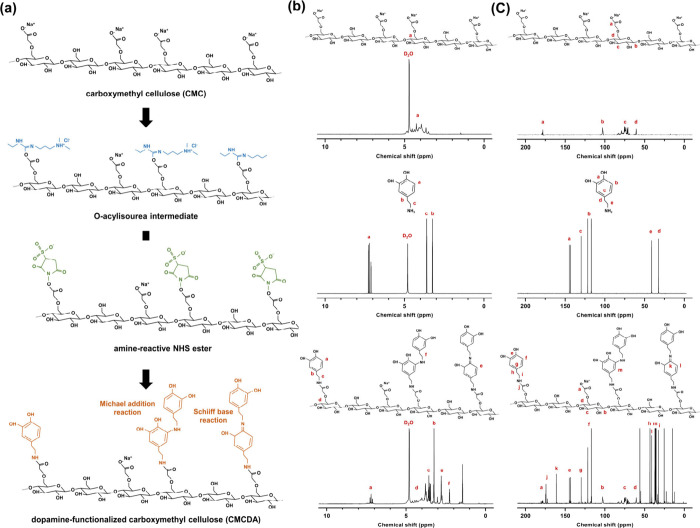
Synthesis and NMR Characterization of CMCDA. (a) Reaction scheme of EDC/NHS-mediated DA grafting onto CMC. (b) ^1^H NMR spectra of CMC, DA, and CMCDA. (c) ^13^C NMR spectra of CMC, DA, and CMCDA.

To verify the proposed molecular structure, multidimensional NMR spectroscopy was performed. The one-dimensional ^1^H NMR spectra (Figure 1b) show signals from the CMC backbone between 3.4 and 5.1 ppm, corresponding to the AGU.^40^ For DA, aromatic protons appear at 7.1–7.3 ppm, which slightly shifted compared to the literature reported by Li et al,^41^ while side-chain CH_2_-NH_2_ and Ar-CH_2_-CH_2_-NH_2_ resonate at 3.2 and 3.6 ppm, respectively.^42^ These DA-derived signals were retained in CMCDA, confirming successful grafting. Additional resonances at 3.1–3.5 ppm are assigned to CH adjacent to enamine groups formed *via* Schiff base reactions, supported by HSQC correlations to a ^13^C signal at 43 ppm (Figure 2). Signals at 2.3–2.8 ppm, together with HSQC correlations to carbons at 30–36 ppm, are attributed to CH_2_ groups formed through Michael 1,4-addition. Based on previous studies on DA-grafted CMC, their ^1^H NMR spectra also showed newly emerged signals in the ranges of 3.1–3.5 ppm and 2.3–2.8 ppm.^27,43^ However, these studies did not thoroughly investigate the detailed and complete molecular structures underlying these peaks. This study fills this gap by providing comprehensive structural elucidation. Furthermore, new peaks at 1.4–1.7 ppm, along with corresponding ^13^C signals at 10–20 ppm, which may originate from partial oxidation of DA to polydopamine-like cyclohexadienone/indoline structures of polydopamine groups upon air exposure along with two-dimensional NMR analysis based on the previous studies.^44,45^

**Figure 2:**
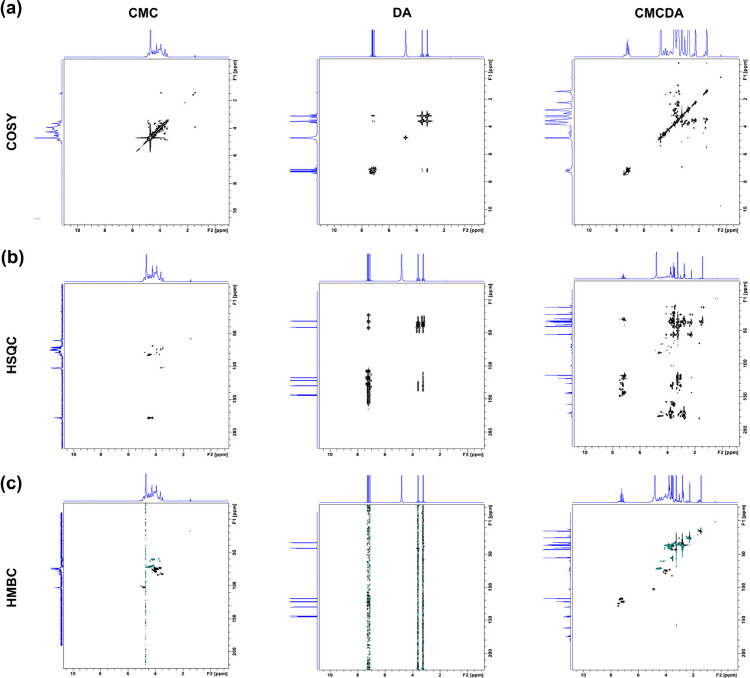
Two-dimensional NMR spectra of CMC, DA, and CMCDA: (a) COSY spectra; (b) HSQC spectra; (c) HMBC spectra.

In the ^13^C NMR spectra (Figure 1c) with DEPT-90 and DEPT-135 analysis (Figure S1), CMC exhibits carboxyl signals at 177.8–180 ppm as previously assigned,^40,46^ which are also present in CMCDA, indicating that DA conjugation does not fully consume all carboxyl groups. Carboxymethyl C-6 appears at 60.5 ppm, ring carbons (C-2–C-5) at 68.2–84.6 ppm, and anomeric C-1 at 100–105 ppm, corresponding to the previous assignment presented by Idström et al.^47^ DA showed aromatic O-substituted tertiary carbons (Ar-C-O) at 143–145 ppm, ipso-tertiary carbons at 130 ppm (both absent in DEPT90/135), and aromatic CH carbons at 117–121 ppm (positive in DEPT90/135). The side-chain CH_2_ carbons at 32 and 41 ppm (negative in DEPT135) correspond to Ar-CH2- and CH_2_-NH_2_ based on the previous studies, respectively.^42,48^ For CMCDA, in addition to all the characteristic CMC and DA peaks, new resonances at 172–175 ppm were assigned to amide C=O, corresponding to the similar structure reported by Guo et al,^49^ providing direct evidence of DA conjugation to the CMC backbone. A signal at 161 ppm, together with an HSQC correlation to ^1^H at 3.3 ppm, supports the presence of C=N in Schiff base enamines, while a ^13^C signal at 43 ppm (negative in DEPT135) is attributed to the adjacent CH. A similarly prominent signal has also been observed in related structures reported in previous studies;^49^ however, detailed explanations have been lacking. We elucidated this previously unresolved structure using multidimensional NMR techniques. New peaks at 34–37 ppm, correlating in HMBC with ^1^H at 2.4–2.8 ppm, are consistent with CH_2_ groups formed *via* Michael addition. Finally, the signals at 12, 15, 22, and 25 ppm were ascribed to the oxidized polydopamine segments, in agreement with ^1^H NMR analysis.

Three key parameters were quantified using NMR signal integration: DS for carboxymethylation of the CMC backbone and DS for DA functionalization and amidation conversion. The native CMC exhibited a carboxymethyl DS of 39.52 ± 1.27%. After functionalization, the DS for DA grafting was 16.63 ± 0.59%, and the amidation conversion between the carboxyl and amine groups reached 68.41 ± 0.08%, indicating a high coupling efficiency under EDC/NHS catalysis. In the dopamine–sodium carboxymethyl cellulose hydrogel reported by Chen et al., the highest DS for DA functionalization was 15%,^27^ whereas Zhong et al. reported a maximum value of 13.5%.^50^ These reports suggest that the DS for DA functionalization is generally modest in the existing literature and our DS is slightly higher than most reported values. We expected that a higher DS for DA functionalization and amidation conversion could increase the hydrogel cross-linking density, as more reactive sites are available for oxidative cross-linking. Consequently, the network becomes more compact, which may reduce porosity due to tighter chain packing and more densely formed cross-links. In parallel, the mechanical properties are anticipated to improve because a denser cross-linked network can better withstand external mechanical stresses. This is particularly advantageous for biomedical sensing applications, where the hydrogel must resist stretching and mechanical perturbations during its use.

FTIR and UV–vis spectroscopy further confirmed successful conjugation. In the FTIR spectrum (Figure S2), unmodified CMC exhibited characteristic bands at 3422 cm^–1^ (O–H), 2900 cm^–1^ (C–H), 1620 cm^–1^ (C=O), and 1058 cm^–1^ (C–O), whereas CMCDA displayed additional bands at 1282 cm^–1^ (aromatic C=C overlapping with C=O) and 1570 cm^–1^ (N–H bending), consistent with the previous literature.^27^ In the UV–vis spectrum (Figure S3), CMCDA exhibited DA-related absorption at ∼ 280 nm assigned to the π–π* transition of sp^2^ domains in aromatic ring of it,^51^ which was absent in CMC, along with a slightly blue-shifted peak at 278 nm, confirming the incorporation of catechol groups. Furthermore, the *M*_w_ of pristine CMC and modified CMCDA, as determined by GPC, were 258342 and 274291 g mol^–1^, respectively. The slight increase in *M*_w_ further confirmed the successful grafting of dopamine onto the CMC. Taken together, these results provide strong evidence for the grafting of DA onto the CMC backbone.

### 3.2 Analysis of Oxidative Cross-Linking

After comprehensive structural characterization, oxidative cross-linking was performed by adding NaIO_4_, which converted the grafted DA groups into polydopamine, transforming the uncrosslinked CMCDA into a cross-linked CMCDA’ hydrogel (Figure 3a). It is important to note that the molecular structure of polydopamine formed during oxidative cross-linking is highly complex and remains a subject of ongoing discussion in the literature.^52,53^ Therefore, the cross-linking mechanism is presented as a simplified conceptual model intended to illustrate the formation of an interconnected network through oxidative transformation of catechol groups rather than representing the exact molecular structure of polydopamine.

**Figure 3:**
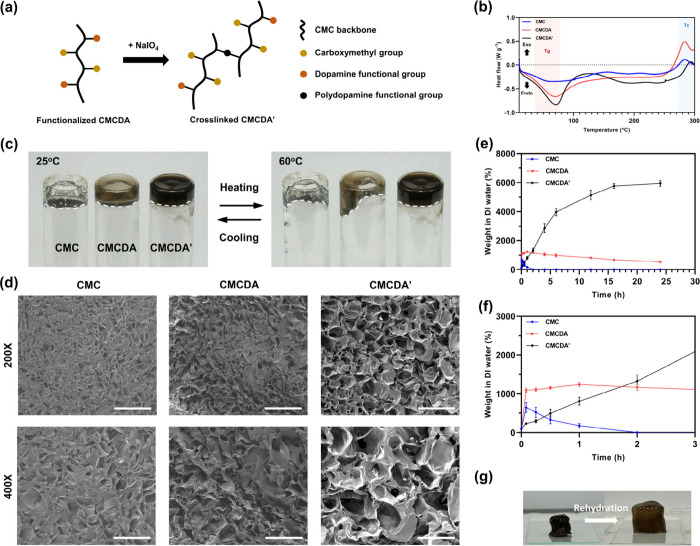
Oxidative cross-linking, adhesion, morphology, and swelling behavior of CMC-based hydrogels. (a) Schematic of NaIO_4_-induced oxidation of dopamine groups in CMCDA to form a cross-linked polydopamine network in CMCDA’. (b) DSC thermograms of CMC, CMCDA, and CMCDA’ from 10 to 300 °C. (c) Macroscopic appearance of CMC, CMCDA, and CMCDA’ at 25 and 60 °C, highlighting the thermal stability of the cross-linked CMCDA’. (d) SEM images of CMC, CMCDA, and CMCDA’ at 200× (scale bar: 400 μm) and 400× magnifications (scale bar: 200 μm). Swelling behavior of CMC, CMCDA, and CMCDA’ in deionized water over (e) 24 h and (f) within 3 h (*n* = 3). (g) Photograph illustrating the rehydration of dried hydrogels in water. Data are shown as mean values ± SD.

To evaluate the effect of functional group engineering on thermal stability, pristine CMC, functionalized CMCDA, and cross-linked CMCDA’ hydrogels were examined at 25 and 60 °C (Figure 3c). At 25 °C, all samples remained in a solid or gel-like state, which was attributed to chain entanglement and extensive hydrogen bonding between the hydroxyl groups.^54^ However, CMC is thermosensitive at 60 °C. The enhanced molecular motion disrupts hydrogen bonding, causing pristine CMC and uncross-linked CMCDA, which lack covalent cross-linking, to melt into flowable liquids. In contrast, the oxidatively cross-linked CMCDA’ hydrogel retained its integrity at elevated temperatures owing to its stable covalent network, highlighting the importance of cross-linking for thermal robustness in biosensing and wearable applications. To further evaluate the effect of dopamine functionalization and oxidative cross-linking on the thermal stability and network integrity of the samples, DSC and rheological temperature ramp analyses were performed, as shown in Figure 3b and Figure S4. Pristine CMC exhibited a glass transition temperature (*T*_g_) at 61.4 °C and a crystallization temperature (*T*_c_) at 282.8 °C, consistent with previous reports.^55,56^ After dopamine functionalization, both *T*_g_ and *T*_c_ increased to 68.9 and 284.1 °C, respectively, indicating enhanced thermal stability, which can be attributed to the catechol groups that strengthened intermolecular interactions and restricted polymer chain mobility.^57,58^ This trend was consistent with the rheological temperature ramp results (25–60 °C), where pristine CMC showed a pronounced decrease in storage modulus (*G*′) of 32.66 ± 3.07% with increasing temperature, while uncrosslinked CMCDA exhibited a much smaller reduction of 13.25 ± 3.19%. Notably, oxidatively cross-linked CMCDA’ showed the highest *T*_g_ and *T*_c_ values, at 70.44 and 293.4 °C, respectively, together with only a 3.72 ± 0.24% change in *G*′, demonstrating that oxidative cross-linking generated a denser and more rigid network that effectively suppressed thermally induced chain relaxation and improved both structural integrity and thermal stability.

SEM and swelling experiments were performed to probe the internal structure and water uptake. The SEM images (Figure 3d) show that CMC and uncross-linked CMCDA possess dense, nearly nonporous morphologies. In contrast, the cross-linked CMCDA’ hydrogels exhibited a more open, highly porous network. We proposed that this phenomenon arises from differences in network stability. When the hydrogel is not cross-linked, the polymer chains are mainly held together by weak interactions such as hydrogen bonding and chain entanglement. During freeze-drying, the network can easily collapse or shrink, resulting in a smaller apparent pore size in the SEM images.^59^ In contrast, after cross-linking, the polymer chains are fixed by chemical bonds, which increases the rigidity of the network and stabilizes the structure. As a result, the original water-filled pores are better preserved during drying, leading to the observation of larger pores in the SEM images. Swelling tests (Figures 3e–g) revealed that the CMCDA’ hydrogel gradually swelled in deionized water, with a continuous increase in mass as the water entered (i) physically, by diffusion into the porous network, and (ii) chemically, *via* interactions with the hydroxyl groups on the CMC backbone and DA moieties. In contrast, CMC and uncross-linked CMCDA, which lack a covalent network, progressively dissolved in water and fully dissolved within 2 h. CMCDA showed initial swelling, followed by collapse, retaining 540% of its original mass after 24 h. These results indicate that functional group modification alone had a limited impact on structural stability, whereas oxidative cross-linking markedly enhanced network integrity, increased swelling capacity, and reduced solubility.

To provide direct evidence for NaIO_4_-induced oxidative cross-linking and the formation of a polydopamine-like structure in CMCDA’ hydrogel, XPS survey spectra and high-resolution C 1s, O 1s, and N 1s spectra were analyzed, as shown in Figure 4. In the survey spectrum of CMC, four characteristic peaks were observed at 287, 498, 534, and 1072 eV, corresponding to C 1s, Na KLL (Auger electron), O 1s, and Na 1s, respectively (Figure 4a).^60–62^ In the high-resolution C 1s spectrum, the peaks were assigned to C–C/C–H at 284.7 eV, C=O/O–C–O at 286.3 eV, C–O at 287.8 eV, and O–C=O at 289.3 eV (Figure 4b).^63–65^ In the O 1s region, peaks corresponding to C–O/C=O/O–H, O–C=O, and COO^–^ were observed at 530.9, 532.62, and 535.43 eV, respectively (Figure 4c).^64,65^ No obvious N 1s signal was detected in the CMC, consistent with the absence of nitrogen in the CMC backbone (Figure 4d). After dopamine grafting, a new N 1s peak appeared at 401 eV in the survey spectrum of CMCDA, confirming the introduction of nitrogen-containing groups (Figure 4e),^66^ while the Na KLL and Na 1s signals disappeared after dialysis. In the high-resolution spectra, the C–N bond was identified at 287.7 eV in the C 1s spectrum and at 401.55 eV in the N 1s spectrum, while the O=C–N bond appeared at 288.9 eV in the C 1s spectrum, 532.5 eV in the O 1s spectrum, and 399.5 eV in the N 1s spectrum (Figure 4f–h).^67–70^ For CMCDA’, the sodium signals reappeared because PBS was used as the solvent (Figure 4i). Similar to CMCDA, the C–N bond in the C 1s and N 1s spectrum was assigned, while the O=C–N bond in the C 1s, O 1s and N 1s spectrum was observed as well (Figure 4j,k). Importantly, a characteristic C–N=C peak emerged at 397.8 eV in the N 1s spectrum, indicating the presence of imine bonds associated with the proposed polydopamine-like structure (Figure 4l).^71,72^ Although the possible molecular structure of CMCDA, shown in Figure 1a, may already contain a small amount of imine linkage formed through the Schiff base reaction, no distinct C–N=C peak was observed in the N 1s spectrum of CMCDA, likely due to its low abundance. In addition, the increased proportion of C=C bonds in the C 1s spectrum further supported the formation of polydopamine-like groups, as polydopamine contains conjugated C=C bonds in nitrogen-containing heterocyclic structures.^73^ The relative percentages of each bonding state and the accurate binding energy results are summarized in Table S1. Overall, these results support the proposed oxidative cross-linking mechanism and further confirm the structure of the dopamine-grafted polysaccharide hydrogel.

**Figure 4:**
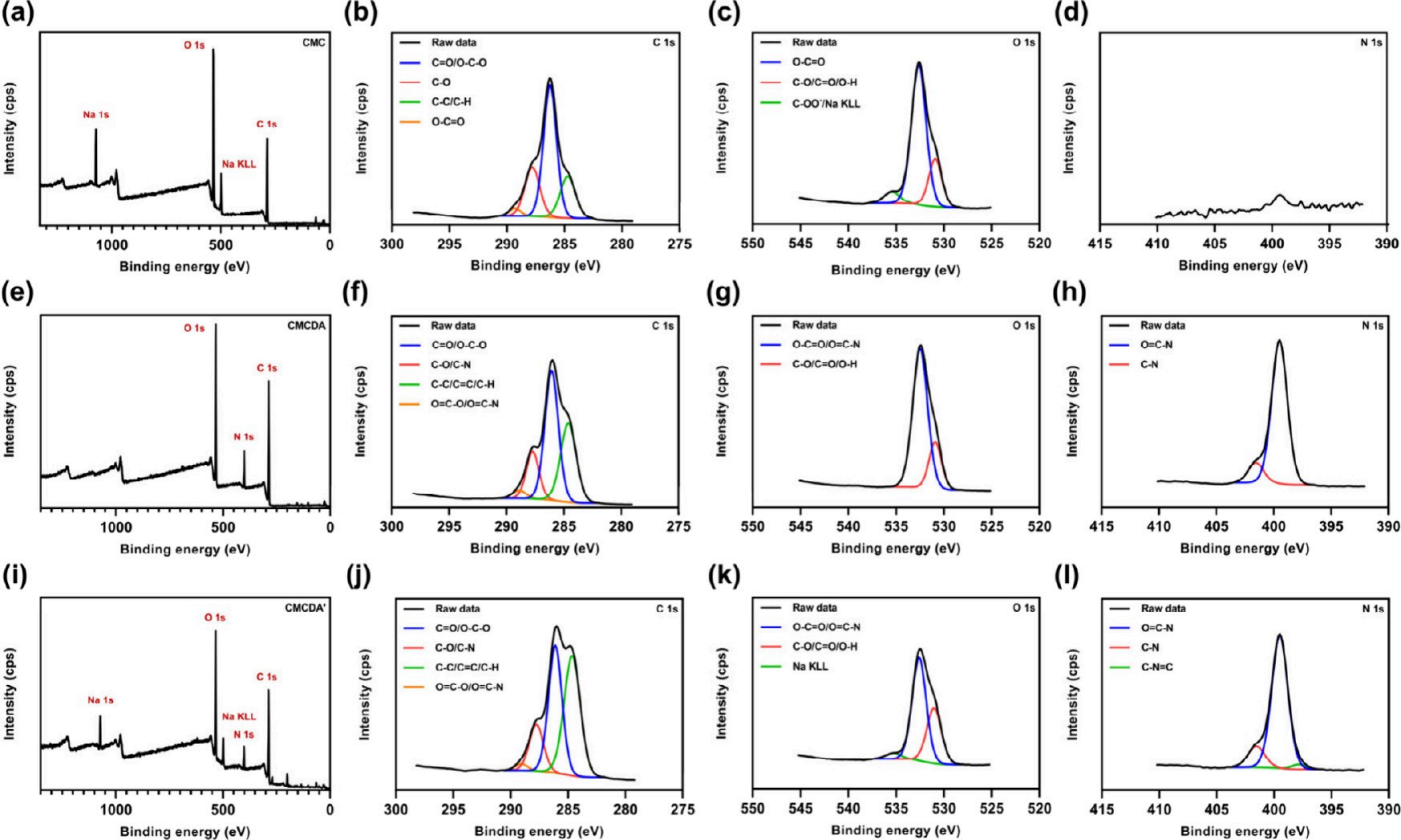
XPS Characterization of CMC, CMCDA, and CMCDA’. From left to right, the panels show the survey spectrum and the high-resolution C 1s, O 1s, and N 1s spectra. From top to bottom, the samples are (a–d) CMC, (e–h) CMCDA, and (i–l) CMCDA’, respectively.

### 3.3 Rheological Analysis

Rheological tests were performed to elucidate the effects of the functional group modification and oxidative cross-linking on the mechanical properties. Many studies on bioinspired dopamine-grafted cellulose-based hydrogels either did not report their mechanical properties or only provide limited rheological data.^27,49,50^ Therefore, we performed rheological measurements as well as tensile and compression tests to quantitatively evaluate the stiffness of our material, thereby offering a more comprehensive characterization of its mechanical properties. The temperature-dependent rheological behavior was examined. At 25 °C (Figure 5a–c), CMC, CMCDA, and cross-linked CMCDA’ all exhibited shear-thinning behavior with similar flow curves, except for a steeper slope for CMCDA’. Power-law fitting (Table 2) showed that CMCDA’ had a lower flow behavior index (*n*) and a higher consistency factor (*K*) than CMC and CMCDA, indicating a higher viscosity and more pronounced shear thinning, which is beneficial for injectability.^74^ In the strain sweep tests, CMCDA’ displayed higher storage (*G*′) and loss (*G*′′) moduli than CMC and CMCDA, whereas those of CMC and CMCDA were comparable. The *G*′–*G*′′ crossover, reflecting the onset of structural breakdown at a given oscillatory strain, revealed strain tolerance in the order of CMCDA > CMCDA’ > CMC. The higher apparent tolerance of uncross-linked CMCDA likely arises from its fluid-like behavior and less-defined gel fracture. All three samples exhibited comparable self-healing behaviors at 25 °C. Besides, From the strain sweep tests, the storage modulus (*G*′) of CMCDA’ hydrogel was determined as 272333 ± 11585 Pa. The *M*_C_ of cross-linked CMCDA’ hydrogel was 5764 g mol^–1^, and the cross-linking density of the CMCDA’ hydrogel was then calculated with measured density of the hydrogel (662.63 kg m^–3^) as 109.86 ± 4.67 mol m^–3^. To facilitate the understanding of the changes in functional group modification and cross-linking, the basic material properties of CMC, CMCDA, and CMCDA’ hydrogels, including the DS for carboxymethylation, DS for dopamine functionalization, amidation conversion, molecular weight, and cross-linking density, are summarized in Table 1.

**Table 1:** Characterizations of CMC, CMCDA, and CMCDA’

sample	DS_carboxymethyl_ (%)	DS_DA_ (%)	amidation conversion (%)	*M*_W_ (g mol^–1^)	*M*_c_ (g mol^–1^)	cross-linking density (mol m^–3^)
CMC	39.52 ± 1.27			258342		
CMCDA		16.63 ± 0.59	68.41 ± 0.08	274291		
CMCDA’					5764	109.86 ± 4.67

**Figure 5:**
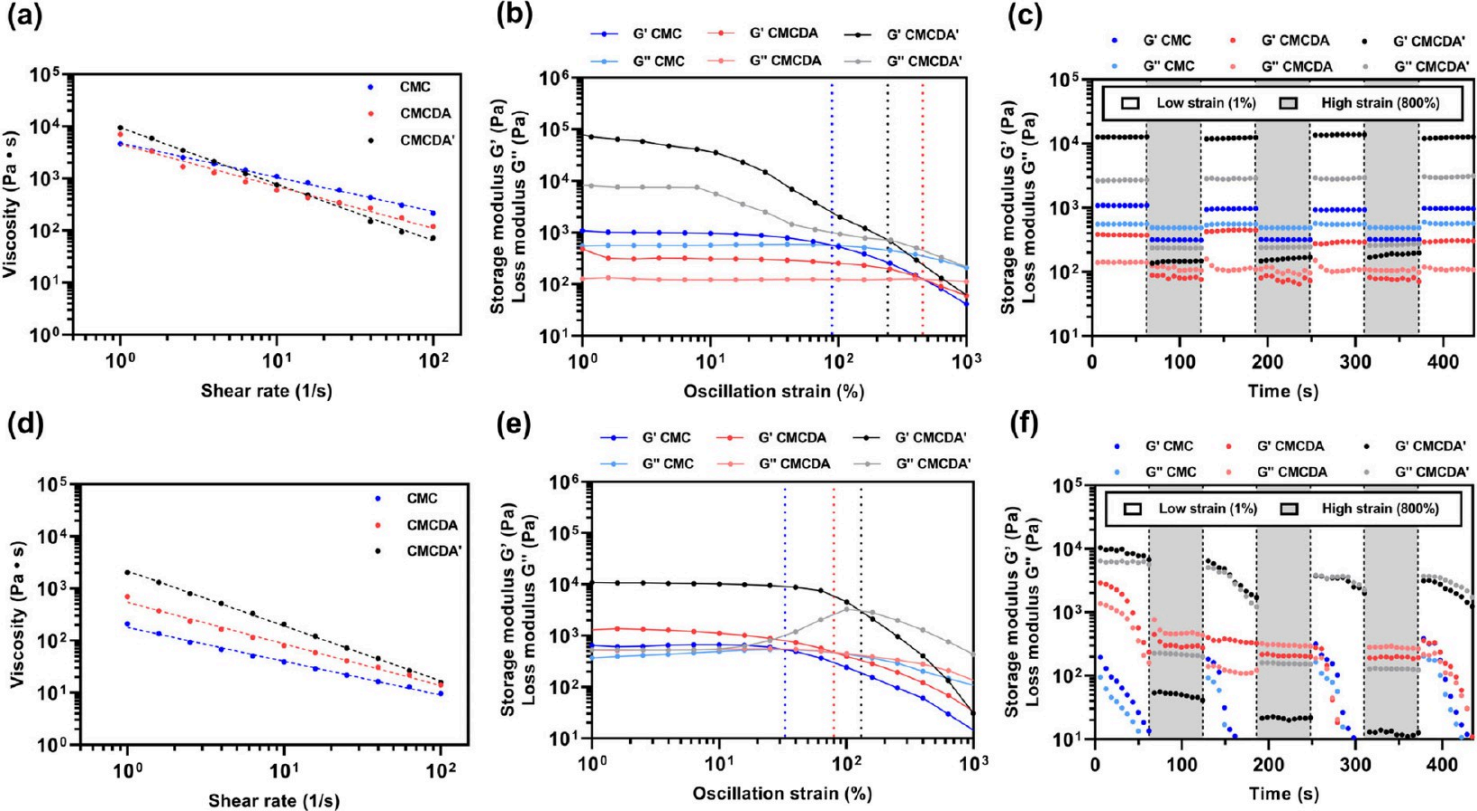
Rheological properties of CMC, CMCDA, and CMCDA’ hydrogels. (a) Viscosity under different shear rates at 25 °C. (b) *G*′ and *G*′′ moduli versus oscillation strain at 25 °C. (c) Step-strain self-healing test (1%/800% strain) at 25 °C. (d) Viscosity under different shear rates at 60 °C. (e) *G*′ and *G*′′ moduli versus the oscillation strain at 60 °C. (f) Step-strain self-healing test (1%/800% strain) at 60 °C.

**Table 2:** Power-Law Fitting Parameters (*n* and *K*) and *R* ^2^ Values for CMC, CMCDA, and CMCDA’ at 25 and 60 °C (*n* = 3)

	temp (°C)	CMC	CMCDA	CMCDA’
*n* value	25	0.342 ± 0.008	0.212 ± 0.037	0.036 ± 0.004
	60	0.352 ± 0.012	0.202 ± 0.024	0.027 ± 0.007
*K* value	25	439.434 ± 25.021	415.137 ± 33.018	823.608 ± 86.221
	60	179.308 ± 18.475	546.135 ± 33.018	2155.262 ± 23.663
*R* ^2^	25	0.9979	0.9751	0.9968
	60	0.9949	0.9928	0.9991

At 60 °C (Figure 5d–f), the differences became more pronounced. All samples exhibited reduced viscosity, but the viscosity at a given shear rate followed CMCDA’ > CMCDA > CMC, demonstrating that both functionalization and cross-linking help maintain viscosity and structural integrity at elevated temperatures, consistent with more distinct *n* and *K* values. Previous studies have also suggested that a high K value with minimal temperature-induced variations may arise from a more robust hydrogel structure, which would be advantageous for biomedical sensing applications and other use scenarios requiring strong resistance to mechanical damage.^37^ Under a strain sweep, the strain tolerance reversed to CMCDA’ > CMCDA > CMC, and in self-healing tests, only CMCDA’ retained relatively stable *G*′ and *G*′′ values after repeated damage, whereas CMC and CMCDA showed unstable moduli with progressive decay over time. These results indicated that oxidative cross-linking significantly reinforced the hydrogel network and enhanced its mechanical robustness.

### 3.4 Mechanical Properties

The cross-linked CMCDA’ hydrogel exhibited outstanding mechanical robustness (Video S1) where substantial tensile deformation was observed. In contrast, unmodified CMC could not form a stable shape or be demolded, indicating that oxidative cross-linking fundamentally altered both the mechanical behavior and morphology of the material. The tensile (Figure 6a) and compressive (Figure 6b) tests directly reflected the bulk mechanical strength. Because of the absence of covalent cross-linking, CMC and CMCDA could not maintain a stable shape and thus could not be demolded for mechanical testing. In contrast, the CMCDA’ hydrogels were easily demolded into well-defined shapes for tensile (Figure 6c) and compressive (Figure 6e) measurements, confirming their stretchable and compressible nature. In addition, the CMCDA’ hydrogel exhibited self-healing properties. After being torn under tension or fractured under compression, the damaged surfaces of CMCDA’ could recontact and autonomously heal, allowing the hydrogel to retain a measurable degree of stretchability and compressibility. The fracture stresses for the tensile and compressive tests were 17.78 ± 0.39 and 8.48 ± 0.19 kPa in the pristine state, respectively, whereas the tensile and compressive tests were 5.01 ± 0.05 and 4.59 ± 0.14 kPa in the postfracture state, respectively (Figure 6d). As for the tensile and compressive elastic moduli, they were determined to be 14.34 ± 1.63 and 1.76 ± 0.32 kPa in the pristine state, respectively, while the corresponding tensile and compressive elastic moduli were 9.49 ± 0.46 and 0.98 ± 0.08 kPa in the pristine state, respectively (Figure 6f). The higher stress response under tension than under compression, together with the overall softness of the material, indicates that CMCDA’ is more suitable for tensile-mode deformation. Therefore, subsequent strain-responsive electrical measurements were conducted under tensile loading rather than compressive loading, aligning with the intended use in wearable devices.

**Figure 6:**
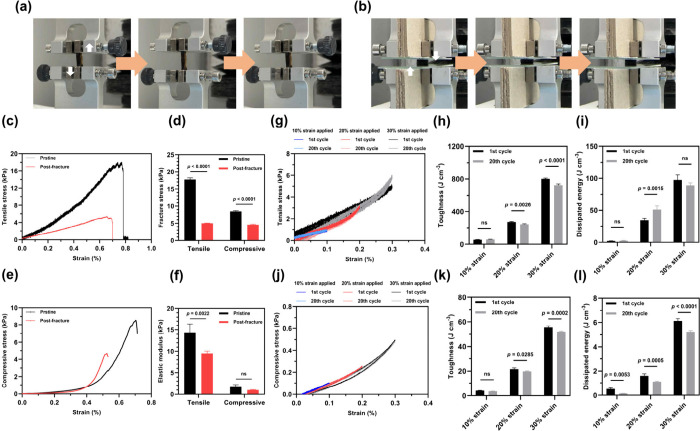
Tensile and compressive mechanical properties of the cross-linked CMCDA’ hydrogel. Photographs of (a) tensile test and (b) compressive test setup. (c) Tensile stress–strain curve of CMCDA’ hydrogels in the pristine state and after fracture. (d) Fracture stress of CMCDA’ under tension and compression hydrogels in the pristine state and after fracture (*n* = 3). (e) Compressive stress–strain curves of CMCDA’ hydrogels in the pristine state and after fracture. (f) Elastic modulus of CMCDA’ under tension and compression in the pristine state and after fracture (*n* = 3). Comparisons between first and 20th cycles for (g) cyclic tensile stress–strain curves at 10, 20, and 30% strain, (h) tensile toughness at different strains (*n* = 3), (i) tensile dissipated energy at different strains (*n* = 3), (j) cyclic compressive stress–strain curves at 10, 20, and 30% strain, (k) compressive toughness at different strains (*n* = 3), and (l) compressive dissipated energy at different strains (*n* = 3). Data are shown as the mean values ± SD. Statistical significance was determined by two-way ANOVA with Tukey’s post hoc test. *p < 0.05, **p < 0.01, ***p < 0.001, ****p < 0.0001; ns, not significant.

To assess the durability of the CMCDA’ hydrogels under repeated deformation relevant to wearable use, cyclic tensile and compressive tests were performed for 20 cycles at 10%, 20%, and 30% strains. In tension, nearly overlapping loading–unloading curves at 10% strain indicated predominantly elastic behavior with good recoverability. At 20% and 30% strain, the hysteresis loops widened, and slight stress softening appeared by the 20th cycle (Figure 6g). The tensile toughness increased with strain but decreased from the first to the 20th cycle at 20% and 30% strain, suggesting mild network weakening under large repeated stretching (Figure 6h). In contrast, the dissipated energy increased markedly with strain and was comparable to or higher in the 20th cycle, particularly at 20% strain, implying enhanced energy loss probably *via* internal friction,^75^ chain slippage,^76^ and viscous processes (Figure 6i).^77^ These irreversible mechanisms reduce the unloading stress more than the loading stress, enlarging the hysteresis area even as the total input energy decreases. At 30% strain, the dissipated energy showed a slight, nonsignificant decrease after cycling, likely owing to the pronounced reduction in toughness and smaller loading–unloading area.

Under compression, the first and 20th stress–strain curves nearly overlapped up to 30% strain, demonstrating excellent structural recovery (Figure 6j). The compressive toughness also increased with strain and showed only minor reductions after 20 cycles, with a noticeable decrease at 30% strain (Figure 6k). The dissipated energy in compression was lower than that in tension but still increased with strain and remained high after cycling at all strain levels (Figure 6l). Overall, the hydrogels retained high toughness while developing a stable energy-dissipation capability under repeated tensile and compressive loading, supporting their long-term mechanical reliability for soft biomedical and wearable applications. To our knowledge, there are currently no systematic studies investigating the property changes of biomimetic cellulose-based hydrogels before and after cross-linking. Although many biomimetic hydrogels have been developed for sensing applications,^78–80^ fundamental mechanistic studies, particularly regarding cross-linking-induced structure–property relationships, remain limited. Such a foundational understanding is crucial for the future design of hydrogels that more closely mimic human tissue while also exhibiting strong bioadhesive performance.

On the whole, the enhanced mechanical performance of the CMCDA’ hydrogel can be understood from a structure–property perspective. Oxidative cross-linking of dopamine groups generates a covalently interconnected network that restricts polymer chain mobility and improves load transfer throughout the hydrogel matrix. In addition, the porous microstructure observed in the SEM images (Figure 3d) provides additional deformation space, allowing pore compression and polymer chain rearrangement during mechanical loading. This structural feature contributes to the high toughness and strain tolerance of the hydrogel. During cyclic deformation, the pronounced hysteresis and energy dissipation likely originate from multiple mechanisms, including reversible catechol-based interactions, polymer chain slippage within the cellulose backbone, and viscous dissipation associated with water migration in the hydrated network. In this regard, our comprehensive characterization of the physical properties associated with oxidative cross-linking provides a valuable reference for more rational and advanced hydrogel design in subsequent studies.

The adhesive properties of the hydrogels are crucial for skin-mounted biosensing, particularly on irregular tissue surfaces and during long-term measurements. Therefore, lap shear and 180° peel tests were performed using porcine skin. As shown in Figure 7a, the stretching of the skin during detachment indicates the adhesive interaction provided by the hydrogel. In the lap shear test (Figure 7b,c), pristine CMC exhibited negligible adhesion, with a shear strength of only 0.25 ± 0.02 kPa, whereas CMCDA and cross-linked CMCDA’ showed markedly higher values of 1.64 ± 0.64 and 3.07 ± 0.65 kPa, respectively. The enhanced adhesion of CMCDA can be attributed to catechol-mediated interactions with biomolecules on the tissue surface, including hydrogen bonding, π–π interactions, Michael addition, and Schiff base reactions. Although CMCDA and CMCDA’ did not differ significantly, only CMCDA’ showed a significant increase compared with pristine CMC, likely because oxidative cross-linking improved the cohesive strength and mechanical integrity of the hydrogel network, thereby reducing cohesive failure during detachment. A similar trend was observed in the 180° peel test (Figure 7d,e), where the interfacial toughness of CMC, CMCDA, and CMCDA’ increased from 1.00 ± 0.55 to 13.31 ± 3.60 and 35.87 ± 6.42 N m^–1^, respectively. Unlike the lap shear test, CMCDA’ showed a significant improvement over CMCDA, highlighting the importance of oxidative cross-linking in resisting peeling-induced stress at the skin interface. As shown in Figure 7f, CMCDA’ remained firmly attached to wet porcine skin even under stretching, bending, and twisting, demonstrating robust adhesion under sweat-mimicking dynamic conditions. Moreover, CMCDA’ also adhered well to various organic and inorganic substrates, including glass, wood, metal, plastic, and skin (Figure 7g). As illustrated in Figure 7h, the strong adhesion of CMCDA’ originates from the combined effects of oxidized polydopamine-like groups and hydroxyl groups on the cellulose backbone, which can form multiple noncovalent interactions with the skin surface, including hydrogen bonding, π–π interactions, and cation−π interactions, as well as covalent interactions through Michael addition and Schiff base reactions. These synergistic interactions provide a robust basis for wearable bioelectronic applications.

**Figure 7:**
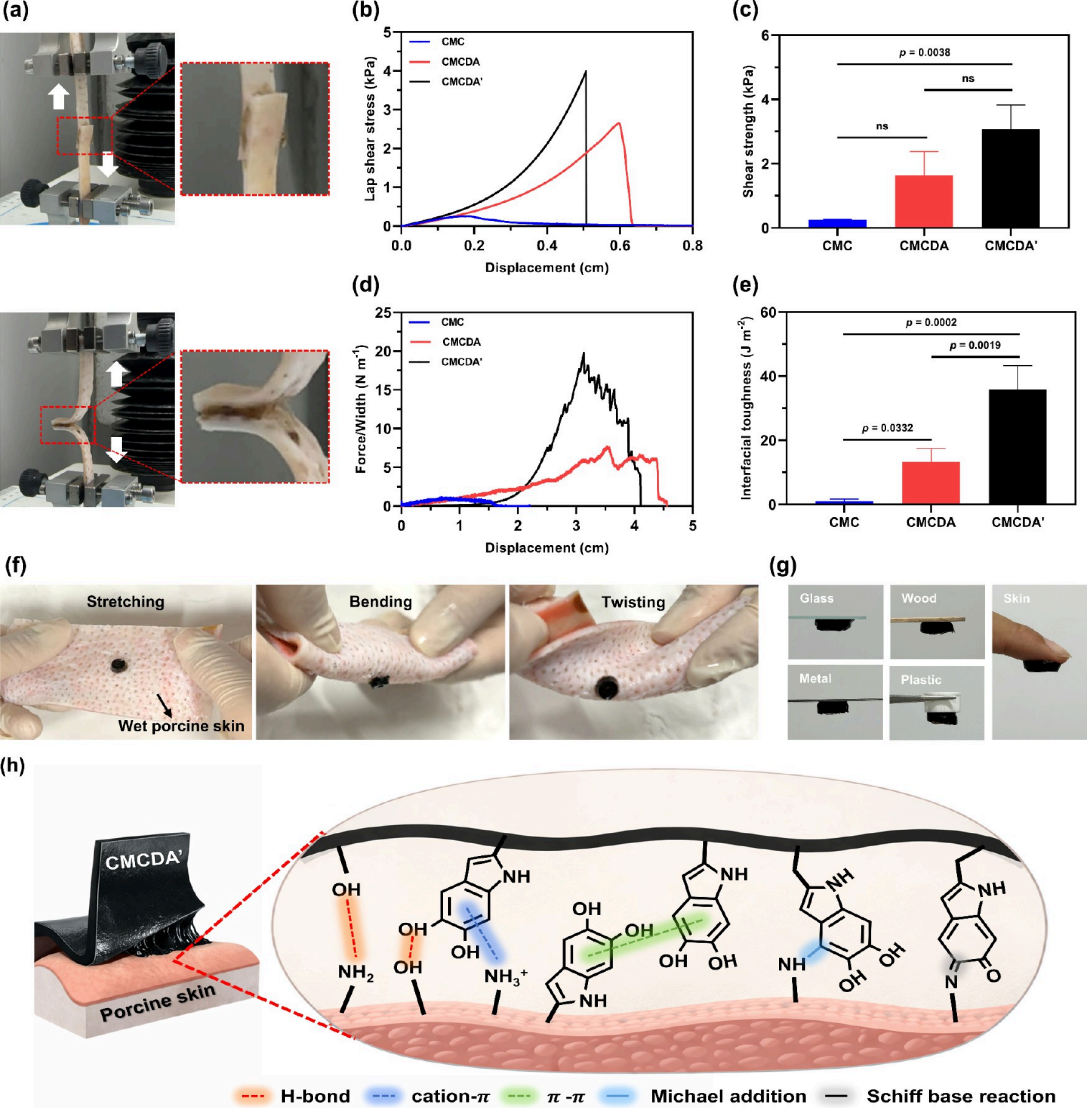
Assessment of adhesion performance of hydrogels. (a) Illustrations of the lap-shear (top) and 180° peel test (down). (b) Stress–strain curves of lap shear test of hydrogel’s adhesion to porcine skin (*n* = 4). (c) Average lap shear strength of the hydrogels to porcine skin tissue (*n* = 4). (d) The measured peeling forces per width of the hydrogel’s adhesion to porcine skin (*n* = 4). (e) Average interfacial toughness of the hydrogels to porcine skin tissue (*n* = 4). (f) Adhesion of the hydrogel to porcine skin under stretching, bending and twisting. (g) Photographs showing strong adhesion of the CMCDA hydrogel on glass, wood, metal, plastic, and skin. (h) Mechanisms of adhesion of CMCDA’ hydrogel on skin tissue. Data are shown as the mean values ± SD. Statistical significance was determined by two-way ANOVA with Tukey’s post hoc test. *p < 0.05, **p < 0.01, ***p < 0.001, ****p < 0.0001; ns, not significant.

### 3.5 Electrochemical Properties

After establishing that functional group modification and cross-linking enhanced the physical properties, the cross-linked CMCDA’ hydrogel with robust mechanics and stable morphology was further converted into an ionically conductive material. The original solvent was replaced with a saturated NaCl solution, which introduced high concentrations of sodium and chloride ions to yield a conductive CMCDA’ hydrogel for electrochemical characterization. As shown in Figure 8a, the hydrogel completed the electrical circuit and lit the LED. Cutting the hydrogel interrupted the circuit and extinguished the LED, whereas the simple apposition of the cut surfaces enabled self-healing and circuit restoration. To evaluate its suitability for long-term biosensing, conductivity was monitored for one week in a moist environment. The impedance spectra as a function of frequency showed negligible changes over time (Figure 8b), and the calculated conductivity remained within 5–10 S m^–1^ (Figure 8c), as determined from the slope of impedance versus hydrogel length using a custom mold (Figure 8d–g). These results demonstrated the excellent long-term ionic conductivity of the CMCDA’ conductive hydrogel. Specifically, Qing et al. investigated how NaCl concentration influences the conductivity of conductive hydrogels.^81^ In our work, we used a saturated NaCl solution at 25 °C (∼5.4 M), which falls within the concentration range reported in their study. Although extremely high ion concentrations may promote ion clustering/ion pairing and thus lead to reduced conductivity,^82^ our hydrogel still meets or even exceeds the conductivity of comparable polyelectrolyte hydrogels.^83^ Notably, Alarcón-Segovia et al. also reported hydrogel electrolytes prepared from cellulose-derived starch and NaCl solution for electrophysiological monitoring.^84^ In comparison, our CMCDA’ conductive hydrogel exhibits a higher conductivity. Moreover, their study did not address the long-term challenges of conductive hydrogels for biosensing, focusing only on monitoring within 8 h, whereas our CMCDA’ conductive hydrogel not only retains low-cost and eco-friendly advantages but also offers bioinspired bioadhesive properties, thereby overcoming key limitations of existing conductive hydrogels for long-term wearable bioelectronics and achieving improved conformal contact on irregular surfaces.

**Figure 8:**
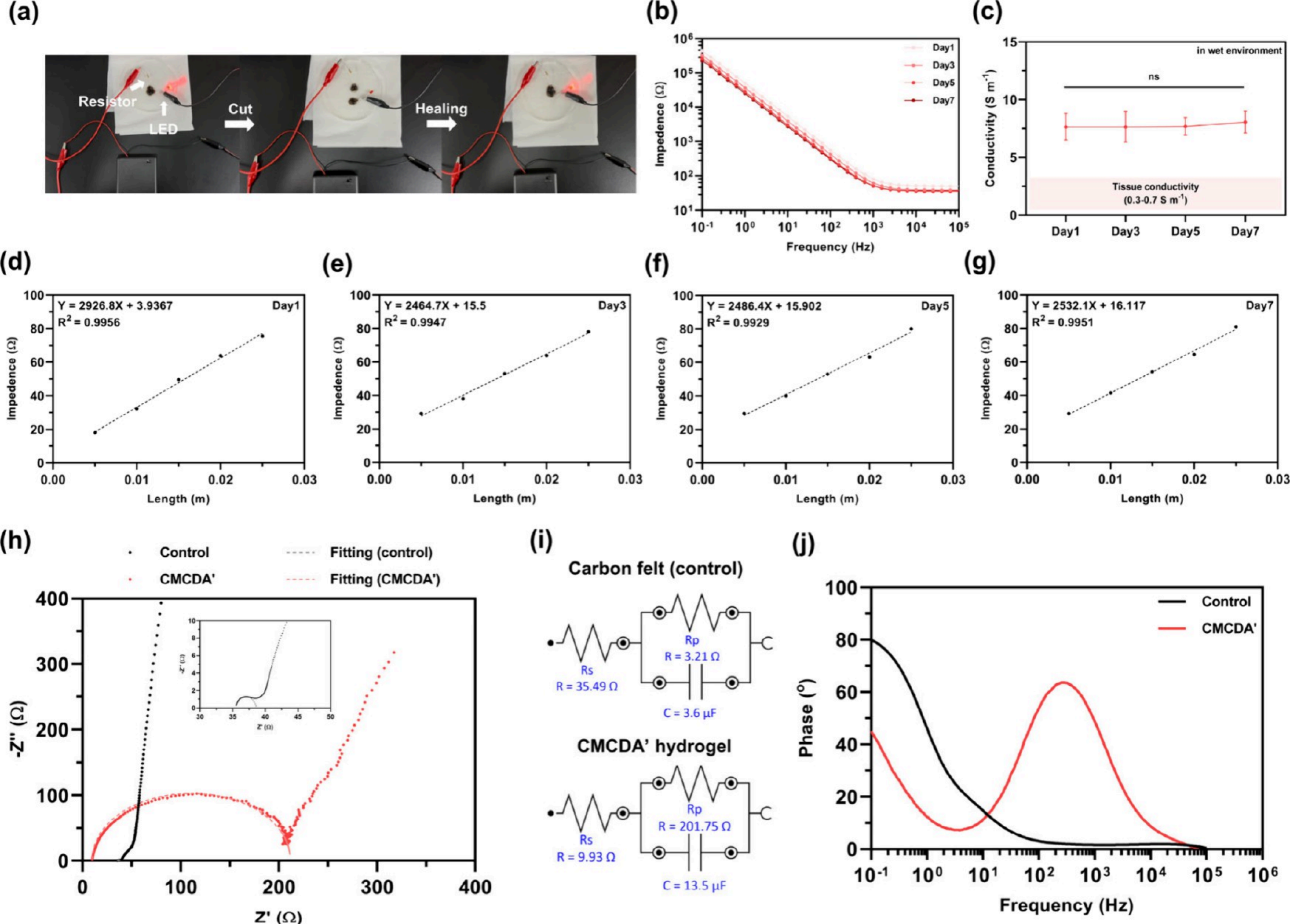
Electrochemical properties of conductive CMCDA’ hydrogel. (a) Photographs of LED circuit showing intact, cut, and self-healed CMCDA’ hydrogel conductor. (b) Impedance spectra of CMCDA’ hydrogel over 7 days. (c) Conductivity of CMCDA’ hydrogel as a function of storage time (*n* = 3). (d–g) Impedance versus hydrogel length used to calculate conductivity on days 1, 3, 5, and 7 (*n* = 3). (h) Nyquist plots of carbon felt (control) and carbon felt/CMCDA’ hydrogel. (i) Equivalent RC circuit models for control and CMCDA’ hydrogel systems. (j) Phase-frequency plots of control and CMCDA’ hydrogel. Data are shown as the mean values ± SD. Statistical significance was determined by two-way ANOVA with Tukey’s post hoc test. *p < 0.05, **p < 0.01, ***p < 0.001, ****p < 0.0001; ns, not significant.

Nyquist plots were then measured to probe the ion transport (Figure 8h). In the control, carbon felt in saturated NaCl, sodium ions and chloride ions accumulated at the electrode/electrolyte interface, forming an electric double layer and producing a small semicircle indicative of a low polarization (charge-transfer) resistance (*R*_p_) and facile ion motion in the electrolyte channels of the felt.^85^ When the CMCDA’ conductive hydrogel was loaded onto the carbon felt, a larger semicircle appeared, reflecting the increased electrolyte transport resistance as the hydrogel partially filled the porous pathways and forced the ions to migrate through its internal porous network. RC circuit fitting of the Nyquist plots (Figure 8i) revealed that *R*_p_ increased from 3.21 Ω (control) to 201.75 Ω (CMCDA’ hydrogel), whereas the solution resistance (*R*_s_) decreased from 35.49 to 9.93 Ω, likely due to the high water content and enhanced ion mobility within the hydrogel.^86^ This combination of low *R*_s_ for efficient signal transmission and high *R*_p_ to suppress Faradaic reactions and polarization is advantageous for ECG biosensing.^87,88^ Phase-frequency plots further showed that carbon felt alone exhibited a typical single-time-constant Randles interface,^89^ whereas the CMCDA’ hydrogel introduced an additional relaxation peak between 100 and 1000 Hz (Figure 8j). This second time constant arises from the additional polymer interface, where ions accumulate at the electrode/hydrogel/electrolyte boundaries, leading to Maxwell–Wagner-type interfacial polarization and a characteristic mid- to high-frequency peak.^90^ To clarify whether the conductivity mainly originated from the absorbed electrolyte or from the intrinsic properties of the polymer network, a nonconductive CMCDA’ hydrogel was prepared using DI water as the solvent. Its impedance spectra and conductivity were then measured (Figure S7). Compared with the conductive CMCDA’ hydrogel prepared with NaCl solution, the nonconductive hydrogel exhibited a much higher impedance and a very low conductivity of only 0–0.2 S m^–1^, which is lower than the conductivity range of biological tissues (0.3–0.7 S m^–1^).^91^ These results indicate that the high conductivity of the conductive CMCDA’ hydrogel primarily originates from the NaCl electrolyte rather than from the polymer network itself, supporting its role as an ionic conductive interface for biomedical applications.

### 3.6 Biosensing Performance and Wearable Bioelectronics

To evaluate the strain-sensing capability of the CMCDA’ hydrogel, it was stretched to determine the relationship between its resistivity and the resultant cross-sectional and microstructural deformation. The hydrogel was elongated by using a universal testing machine, with copper tape electrodes attached to collect electrical signals (Figure S8a). As shown in Figure S8b, the relative resistance change increased with strain, and the GF-strain curve could be divided into three regions (I, II, and III) with distinct GF values. This multistage behavior is attributed to the progressive damage to critical ionic pathways within the porous network. As the strain increases, the key conduction channels are sequentially disrupted, leading to two pronounced transition points and an abrupt increase in resistance.^92,93^

Under 20% cyclic tensile loading for 20 cycles, the change in resistance exhibited minor fluctuations but remained stable overall (Figure S8c), indicating reliable strain responsivity and good cyclic durability. At different strain amplitudes (10%, 20%, and 30%), the resistance response scaled with the strain and the signals recovered to close to their initial values after five cycles at each level (Figure S8d–f). These results demonstrate that the conductive CMCDA’ hydrogel functions as an effective, repeatable strain sensor suitable for detecting multiple bending or stretching events in biosensing applications.

Finally, a wearable bioelectronic platform for ECG recording was constructed by integrating an Arduino UNO microcontroller with a SparkFun AD8232 single-lead heart rate monitor using custom circuitry and code (Figure S9). Figure 9a shows the electrode and ground placements for the ECG measurements at different body sites (wrist, elbow, chest, and neck). Representative one-week ECG recordings obtained with a commercial conductive gel (3M^TM^) and the CMCDA’ hydrogel are compared in Figure 9b,c. The neck produced smaller signal amplitudes, which is reasonable given its weaker pulse vibration. All traces confirm that this low-cost, easily assembled system can reliably capture ECG signals. The signal quality was further evaluated using SNR analysis in Figure 9d. In general, the SNR values were higher for the wrist and chest and lower for the elbow and neck. After one week, the SNR decreased at all sites when the commercial gel was used, underscoring its limitations for long-term biosensing. In contrast, although the SNR obtained with the CMCDA’ hydrogel declined slightly over time, it remained significantly higher than that obtained with the commercial gel at the elbow, chest, and neck, with the most pronounced improvement at the elbow. This enhancement is attributed to the intrinsic bioadhesive properties of the CMCDA’ hydrogel, which maintains intimate contact with curved skin surfaces and thus improves the signal strength and quality. These findings highlight the strong potential of sustainable cellulose-based CMCDA’ hydrogels for use in wearable ECG bioelectronics. In addition, cytocompatibility tests indicated that mouse fibroblast cells (L929) cultured with extracts from the conductive CMCDA’ hydrogel exhibited no evident cytotoxicity (Figure S10). Live/Dead staining showed almost no red (dead-cell) signals, comparable to the control group. Consistently, cell metabolic activity remained normal, and the CMCDA’ hydrogel-treated medium did not compromise L929 cell viability as determined by the CCK-8 assay. Collectively, these results demonstrate that the CMCDA’ hydrogel is biocompatible and provides a sustainable and safe platform for biosensing applications. To better reflect the intended long-term skin-contact application, a direct contact test, morphological observation of L929 cells, and a longer-term viability assessment were conducted for a more comprehensive evaluation of biocompatibility. As shown in Figure S11, the number of L929 cells in both the control and CMCDA’ hydrogel groups increased over time. By day 7, only a very small number of dead cells was observed, likely due to cell crowding, while most cells remained viable and maintained a normal spindle-shaped morphology under optical microscopy. Although the direct contact CCK-8 assay showed significantly lower cell viability in the CMCDA’ hydrogel group than in the control group (Figure S12), the viability remained above 80% on day 7. According to ISO 10993, materials with a cell viability greater than 70% are considered biocompatible. These results indicate that CMCDA’ hydrogel maintains good biocompatibility even under direct contact with cells.

**Figure 9:**
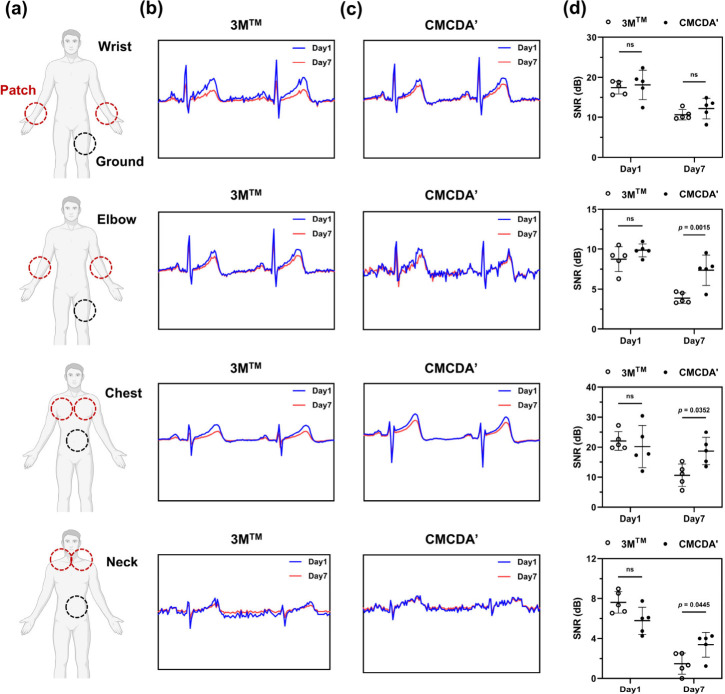
Wearable ECG recording using CMCDA’ hydrogel electrodes. (a) Electrode and ground placement for ECG measurements at the wrist, elbow, chest, and neck. (b) Representative ECG signals on Days 1 and 7 using commercial 3M^TM^ conductive gel. (c) Representative ECG signals on Days 1 and 7 using CMCDA’ hydrogel electrodes. (d) SNR comparison between 3M^TM^ gel and CMCDA’ hydrogel at each body site on Days 1 and 7 (*n* = 5). Data are shown as the mean values ± SD. Statistical significance was determined by two-way ANOVA with Tukey’s post hoc test. *p < 0.05, **p < 0.01, ***p < 0.001, ****p < 0.0001; ns, not significant.

Although CMCDA’ hydrogels exhibit markedly superior mechanical properties and intrinsic bioadhesion compared to commercial conductive gels, they share a key limitation: they cannot be stored in air for long periods because water loss leads to drying and loss of functionality. To address this, future work could adopt the solvent-exchange strategies reported in the literature, replacing the NaCl solution with a glycerol-based electrolyte to enable long-term storage under ambient conditions.^94^ In addition, while saturated NaCl provides high ionic strength and thus good ionic conductivity, excessive ion concentrations can interfere with the oxidative cross-linking of CMCDA and disrupt the cellular osmotic balance, which is undesirable for implantable applications. This issue can be mitigated by incorporating conductive polymers or nanomaterials, such as PEDOT:PSS or graphene, to enhance the conductivity and reduce the reliance on high salt concentrations. Although the present study demonstrates improved signal stability compared with a commercial conductive gel, a systematic comparison with other hydrogel-based bioelectronic interfaces, including CMC-based hydrogels, mussel-inspired adhesive hydrogels, and conductive polymer composite hydrogels (e.g., PEDOT:PSS systems), was beyond the scope of this work. Future studies will further evaluate the relative performance of the CMCDA’ hydrogel against these representative hydrogel systems. Overall, the conductive CMCDA’ hydrogel represents an accessible, low-cost, and environmentally friendly green material that avoids petroleum-derived or toxic components.

## Conclusion

In this study, we developed a dopamine-grafted carboxymethyl cellulose hydrogel, CMCDA, and its oxidative cross-linked counterpart, CMCDA’, as a bioadhesive, ionically conductive platform for wearable ECG electrodes. EDC/NHS coupling achieved a DA DS of ∼16.6% with an ∼68% amidation efficiency. multidimensional NMR, FTIR, and UV–vis spectroscopy jointly confirmed amide formation, Schiff base/Michael addition products, and partially oxidized polydopamine segments on the CMC backbone. Oxidative cross-linking *via* NaIO_4_ generated a stable polydopamine network, giving CMCDA’ superior thermal resistance, a porous microstructure, high swelling capacity, and greatly reduced solubility compared to pristine and uncrosslinked CMCDA. Rheological and mechanical tests revealed higher viscosity, stronger shear thinning, increased moduli, and robust cyclic tensile/compressive performance. After solvent exchange with saturated NaCl, CMCDA’ exhibited long-term ionic conductivity (5–10 S m^–1^), self-healable electrical pathways, a multistage strain-dependent resistance response and great cytocompatibility as a sustainable polysaccharide-based platform for conductive interface of biosensing applications. When integrated into an Arduino-based ECG system, the bioadhesive CMCDA’ hydrogel delivered one-week ECG recordings with higher SNR than a commercial conductive gel, particularly on curved body sites, underscoring its promise for soft, low-cost, and long-term wearable bioelectronics.

## Supplementary Material





## Notes

DEPT90/135, FTIR, and UV–vis spectra of CMC, DA and CMCDA. Temperature ramp tests; table for binding energies and peak areas of XPS. Experimental setup for the measurement of conductivity, Bode plots and Nyquist plots. Impedance spectra and conductivity of nonconductive CMCDA’ hydrogel. Strain-sensing performance of conductive hydrogel. Programming and circuit design of the Arduino-based microcontroller system. Biocompatibility assessment of CMCDA’ conductive hydrogel for extract test and direct contact test (PDF)
Video S1 (MP4)
