# Supplementary material for: Biomimetic Hydrogels with Oxidative Cross-Linking for Ionically Conductive Interfaces in Long-Term Wearable Bioelectronics
Source: Biomacromolecules. 2026 May 8;27(6):3809–27. doi: 10.1021/acs.biomac.6c00288 (PMC13250909; doi:10.1021/acs.biomac.6c00288)
Supplement: Supplementary file 1 [file bm6c00288_si_001.pdf]

Supporting Information

Biomimetic hydrogels with oxidative crosslinking for ionically conductive interfaces in long-term wearable bioelectronics

Kai-Hsiang Chang[a], Wen-Ya Lee[b], and Jiashing Yu[a*]

[a] Department of Chemical Engineering, National Taiwan University, Taipei 10617, Taiwan

[b] Department of Chemical Engineering and Biotechnology, National Taipei University of Technology, Taipei 10617, Taiwan

*Corresponding authors:

Jiashing Yu, E-mail: jiayu@ntu.edu.tw

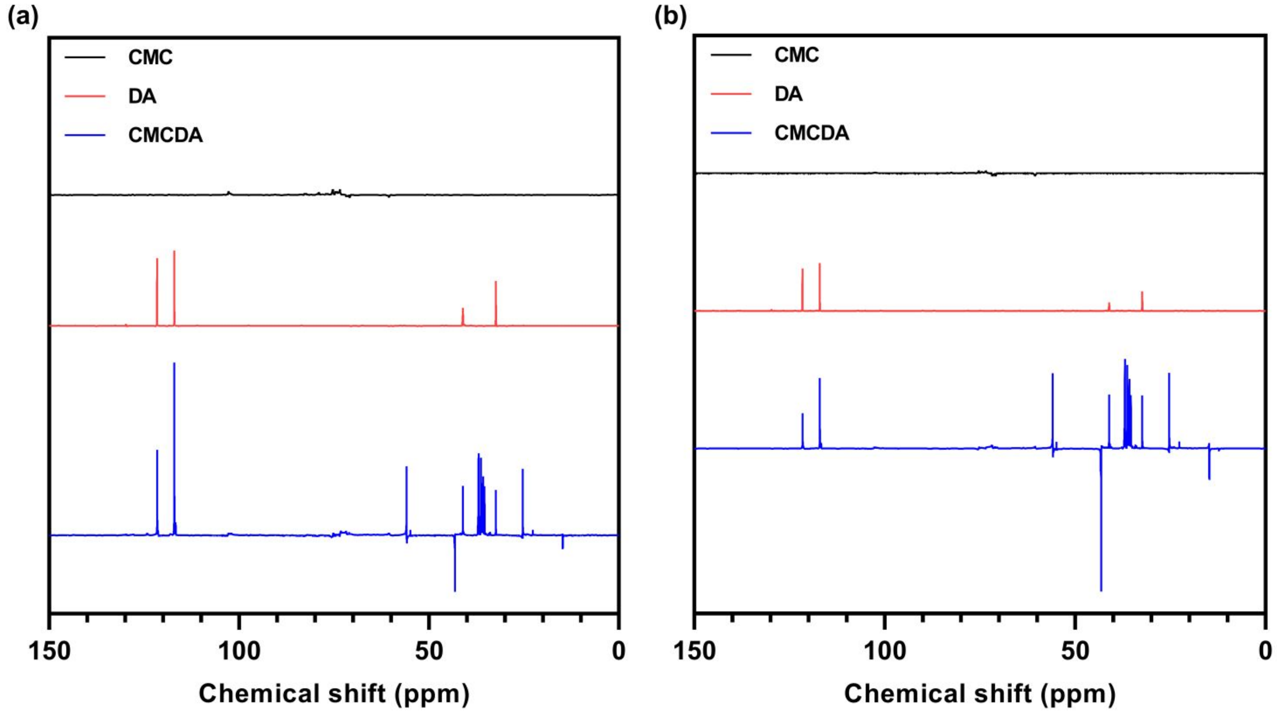

Figure S1. (a) The DEPT90 and (b) DEPT135 spectrum of unmodified CMC, DA and functionalized CMCDA.

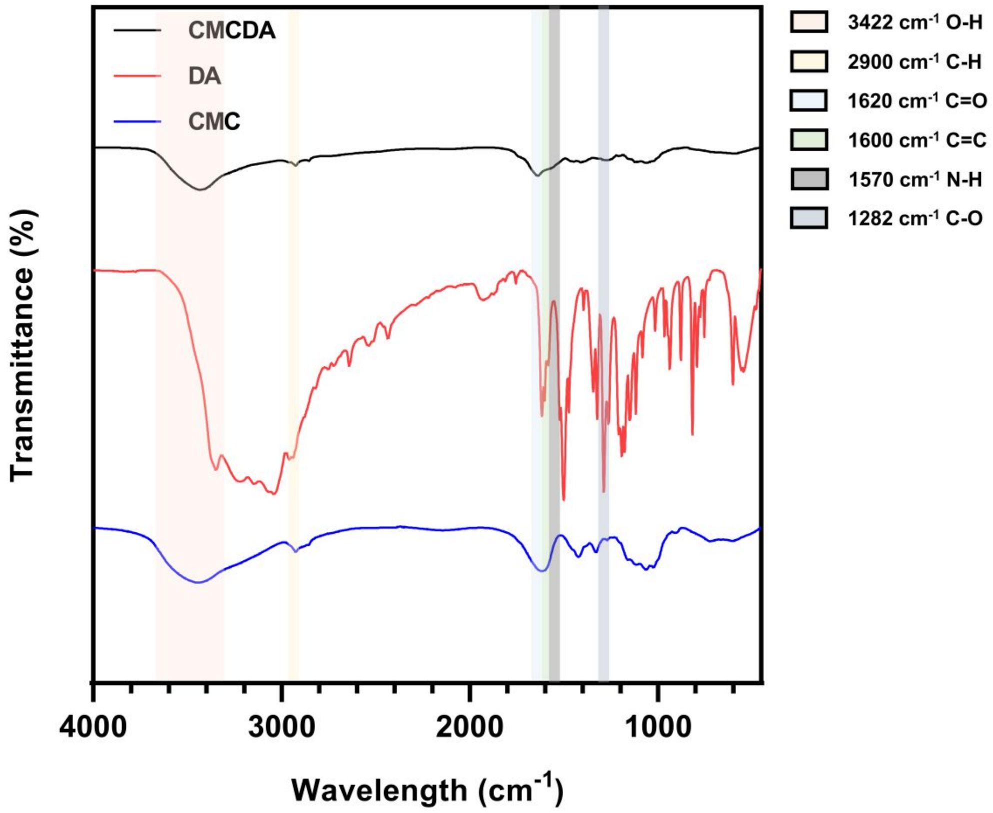

Figure S2. The FTIR spectrum of unmodified CMC, DA and functionalized CMCDA across the wavenumber range of 400 to 4500 cm^{-1}.

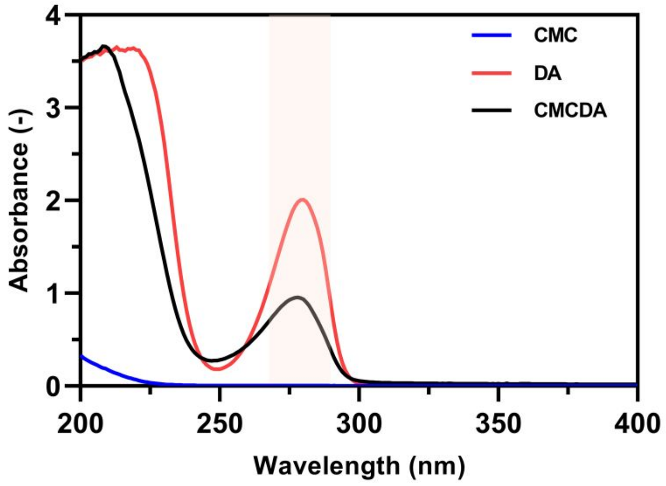

Figure S3. The UV-Vis spectrum of unmodified CMC, DA and functionalized CMCDA with absorbance measured between 200 and 400 nm.

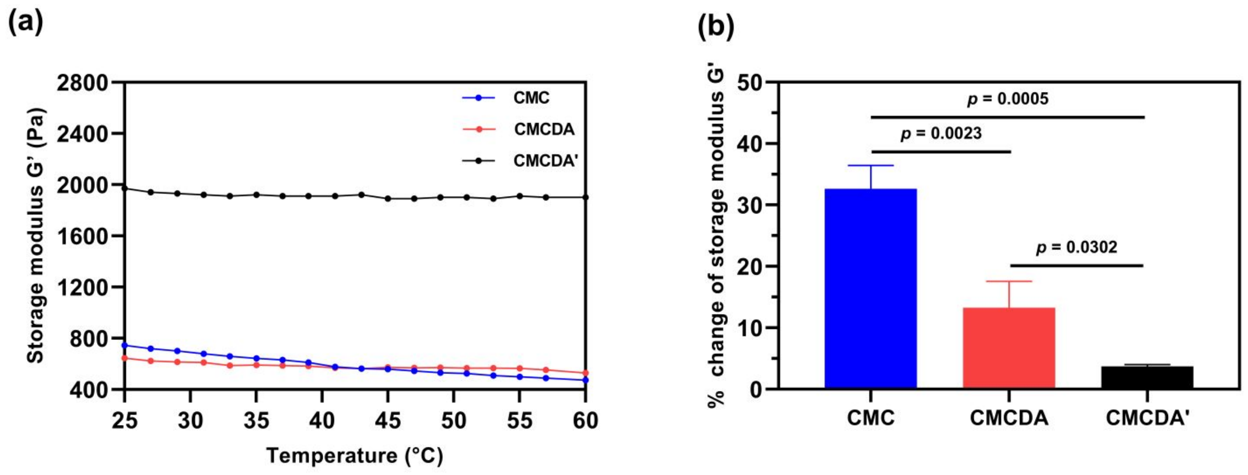

Figure S4. (a) Temperature ramp tests with varying temperature from 25 °C to 60 °C with fixed 0.1% strain and (b) change in storage modulus (G') for CMC, CMCDA, and CMCDA' (n = 3). Data are shown as the mean values ± SD. Statistical significance was determined by two-way ANOVA with Tukey's post hoc test. $*p < 0.05$, $**p < 0.01$, $***p < 0.001$, $****p < 0.0001$; ns, not significant.

Table S1. Binding energies and peak areas of C 1s, O 1s, and N 1s for CMC, CMCDA, and CMCDA'.

Sample	Peak	Binding energy (eV)	Fitted peak area (%)	Assignment
CMC	C 1s	286.3	42.16	C=O/O-C-O
		287.8	23.86	C-O
		284.7	22.11	C-C/C-H

		289.3	11.87	O-C=O
	O 1s	532.6	49.09	O-C=O
		530.9	28.72	C-O/C=O/O-H
		535.4	22.19	C-OO$^-$/Na KLL
CMCDA	C 1s	286.3	42.16	C=O/O-C-O
		287.7	30.30	C-O/C-N
		284.6	11.04	C-C/C=C/C-H
		288.9	19.45	O=C-O/O=C-N
	O 1s	532.5	64.82	O-C=O/O-C=N
		530.9	35.18	C-O/C=O/O-H
	N 1s	399.5	59.39	O=C-N
		401.6	40.61	C-N
CMCDA’	C 1s	286.3	42.16	C=O/O-C-O
		287.7	30.30	C-O/C-N
		284.6	11.04	C-C/C=C/C-H
		288.9	19.45	O=C-O/O=C-N
	O 1s	532.5	64.82	O-C=O/O-C=N
		530.9	35.18	C-O/C=O/O-H
		535.5	24.85	Na KLL
	N 1s	399.5	59.39	O=C-N
		401.6	40.61	C-N
		397.8	29.61	C-N=C

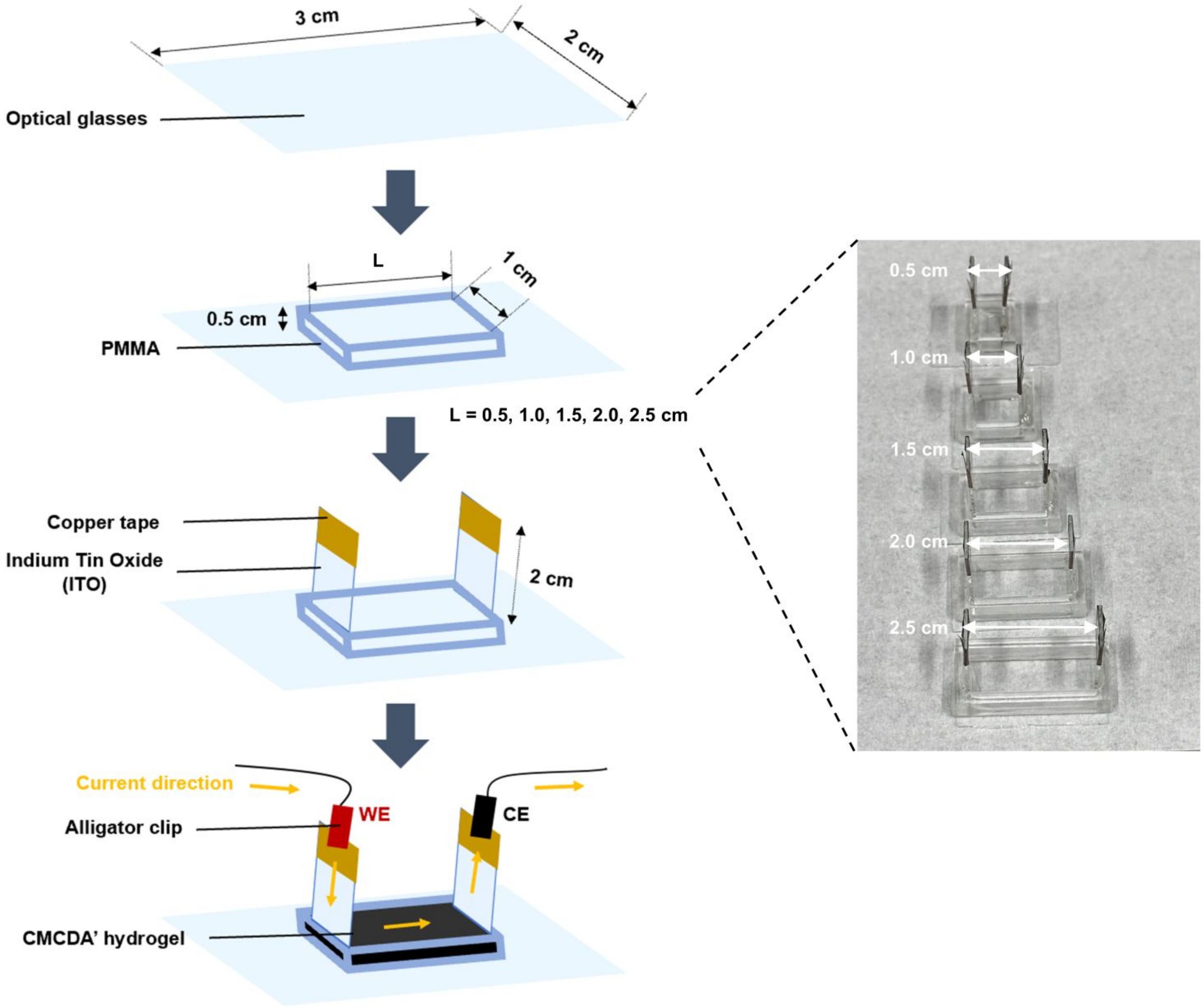

Figure S5. The experimental setup for the measurement of conductivity and Bode plots with custom-fabricated devices.

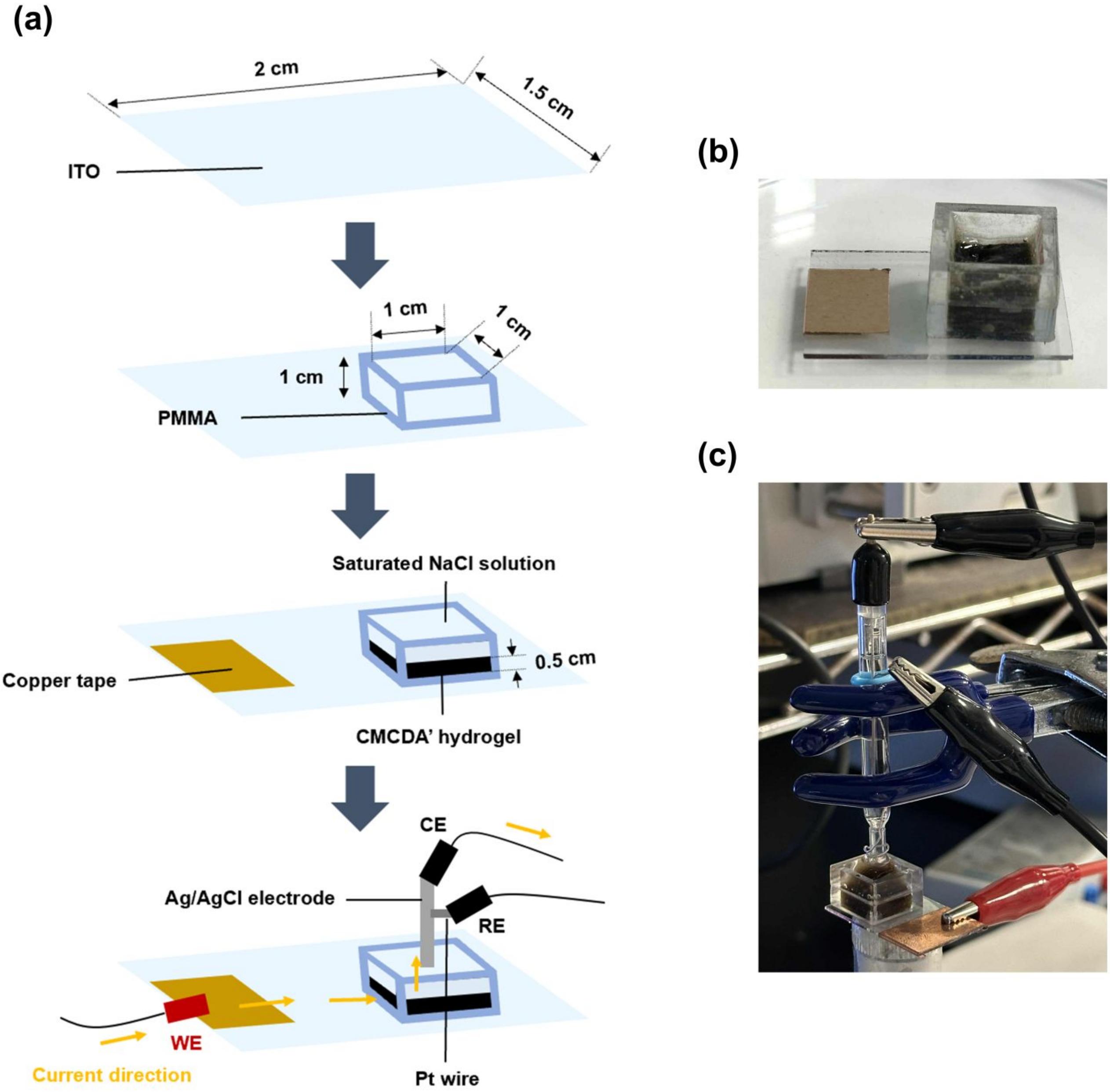

Figure S6. The experimental setup for the measurement of Nyquist plots with custom-made devices.

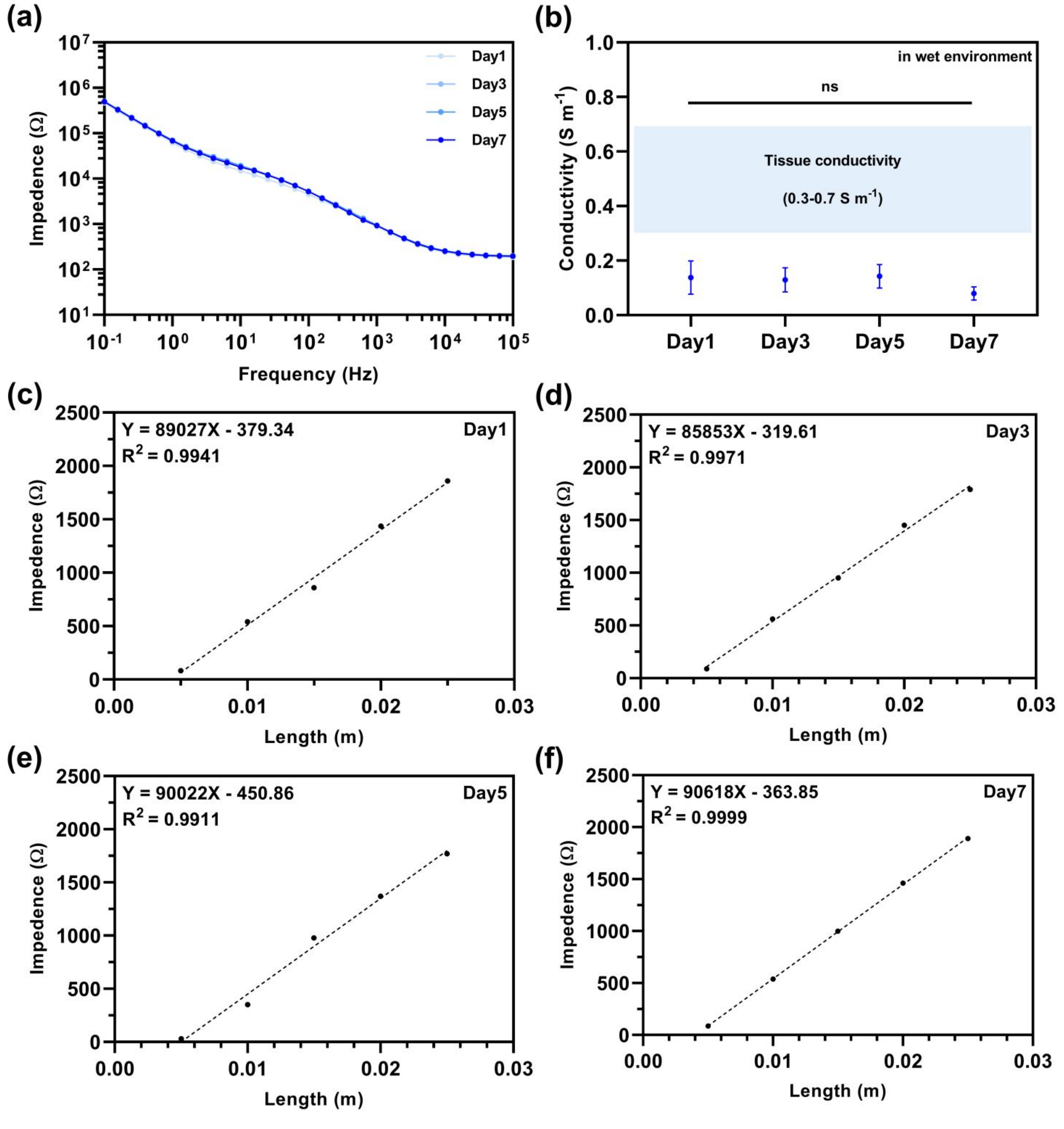

Figure S7. (a) Impedance spectra of non-conductive CMCDA' hydrogel using DI water as the solvent over 7 days. (b) Conductivity of non-conductive CMCDA' hydrogel as a function of storage time (n = 3). (c)-(f) Impedance versus non-conductive CMCDA' hydrogel length used to calculate conductivity on day 1, 3, 5, and 7 (n = 3). Data are shown as the mean values ± SD. Statistical significance was determined by two-way ANOVA with Tukey's post hoc test. *$p < 0.05$, **$p < 0.01$, ***$p < 0.001$, ****$p < 0.0001$; ns, not significant.

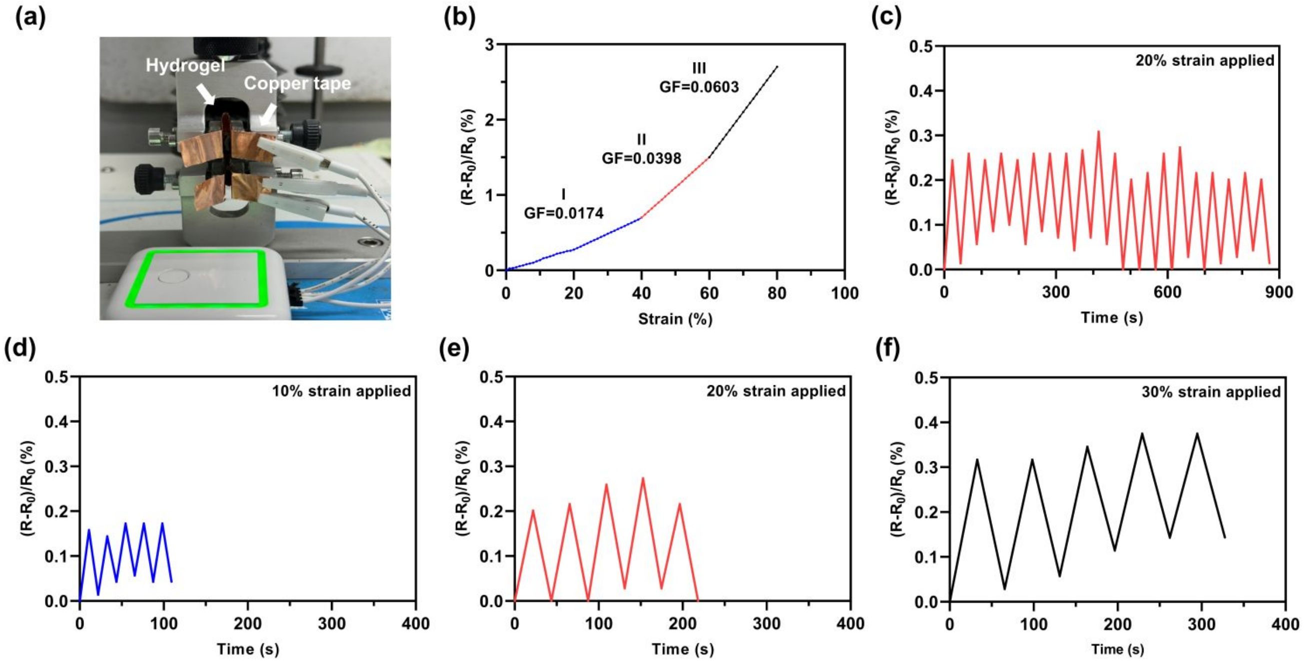

Figure S8. Strain-sensing performance of conductive CMCDA' hydrogel. (a) Tensile strain sensor setup with CMCDA' hydrogel and copper tape electrodes on a universal testing machine. (b) Relative resistance changes versus strain, showing three GF regions (I–III). (c) Cyclic resistance response of CMCDA' under 20% tensile strain for 20 cycles. (d) Cyclic resistance response under 10% tensile strain with 5 cycles. (e) Cyclic resistance response under 20% tensile strain with 5 cycles. (f) Cyclic resistance response under 30% tensile strain with 5 cycles.

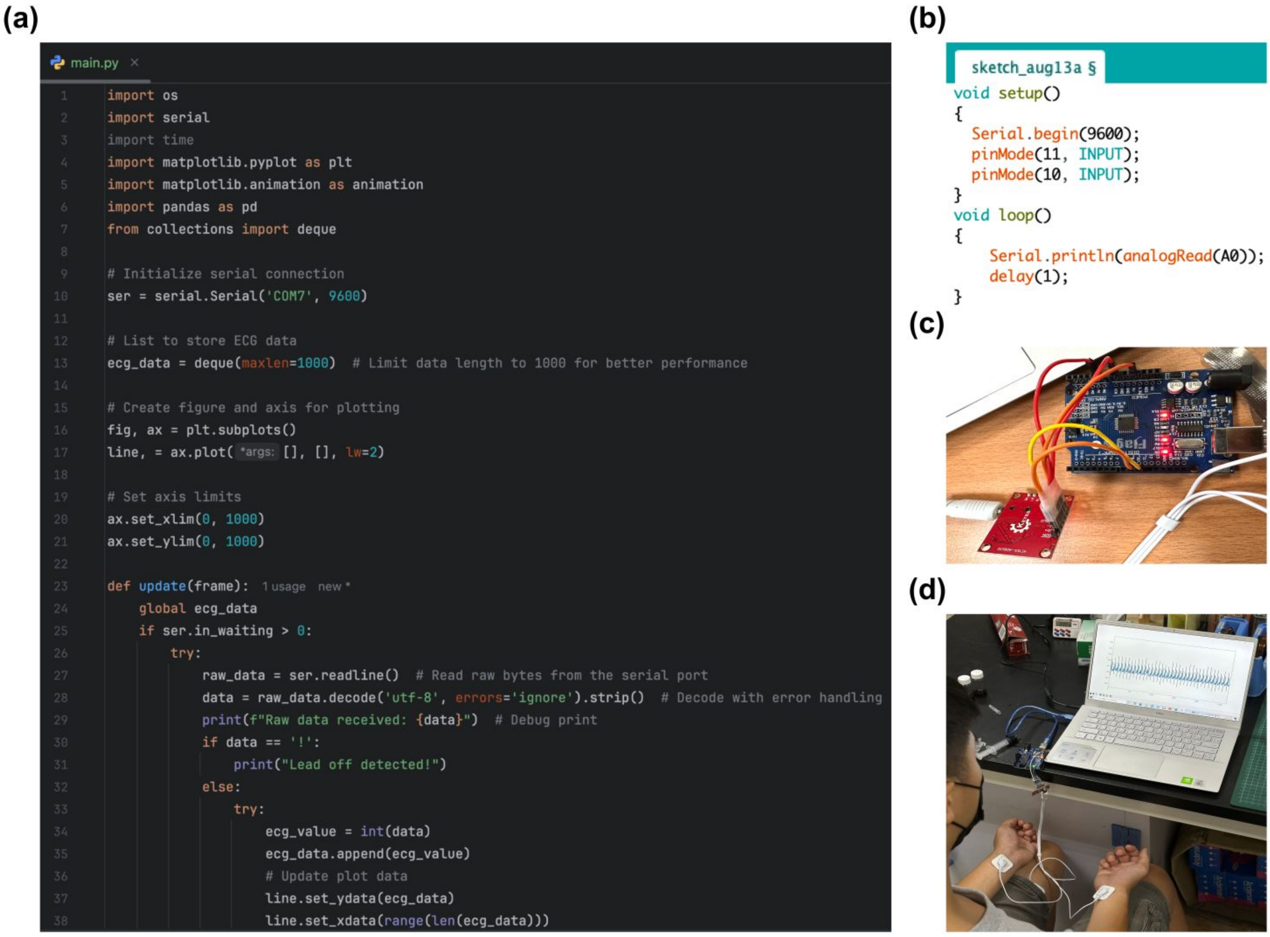

Figure S9. Programming and circuit design of the Arduino-based microcontroller system. (a) Establishment of the Python environment in the PyCharm IDE for data acquisition and processing. (b) Arduino sketch for controlling the board. (c) Circuit diagram integrating the Arduino UNO with the AD8232 SparkFun single-lead heart rate monitor. (d) Photograph of the Arduino UNO–based platform during real-time three-channel ECG monitoring.

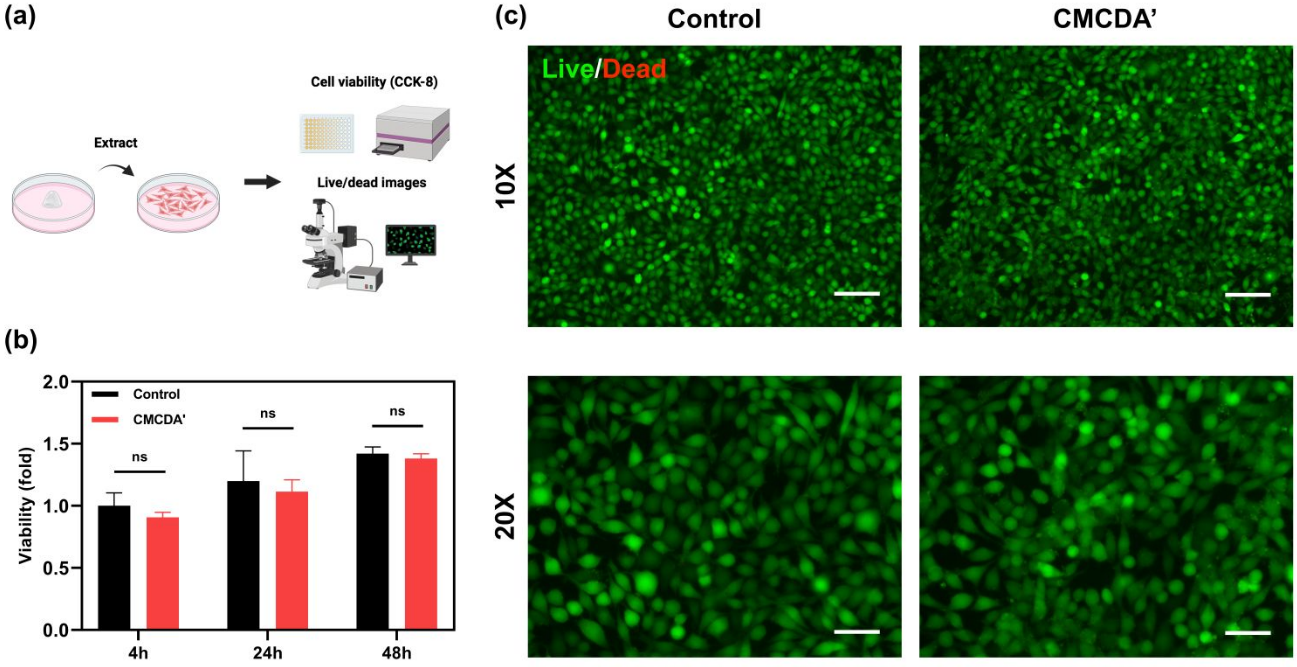

Figure S10. Biocompatibility assessment and cell behavior analysis of the conductive CMCDA' hydrogels. (a) Experimental setup for hydrogel extract in L929 cell culture. (b) CCK-8 assay results of cell viability following exposure to hydrogel extracts for 4, 24, and 48 h. (c) Live/dead staining images of L929 cells after 48 h exposure to hydrogel extracts, acquired at 10X (scale bar: 100 μm) and 20X magnification (scale bar: 50 μm). Data are shown as the mean values ± SD. Statistical significance was determined by two-way ANOVA with Tukey's post hoc test. *$p < 0.05$, **$p < 0.01$, ***$p < 0.001$, ****$p < 0.0001$; ns, not significant.

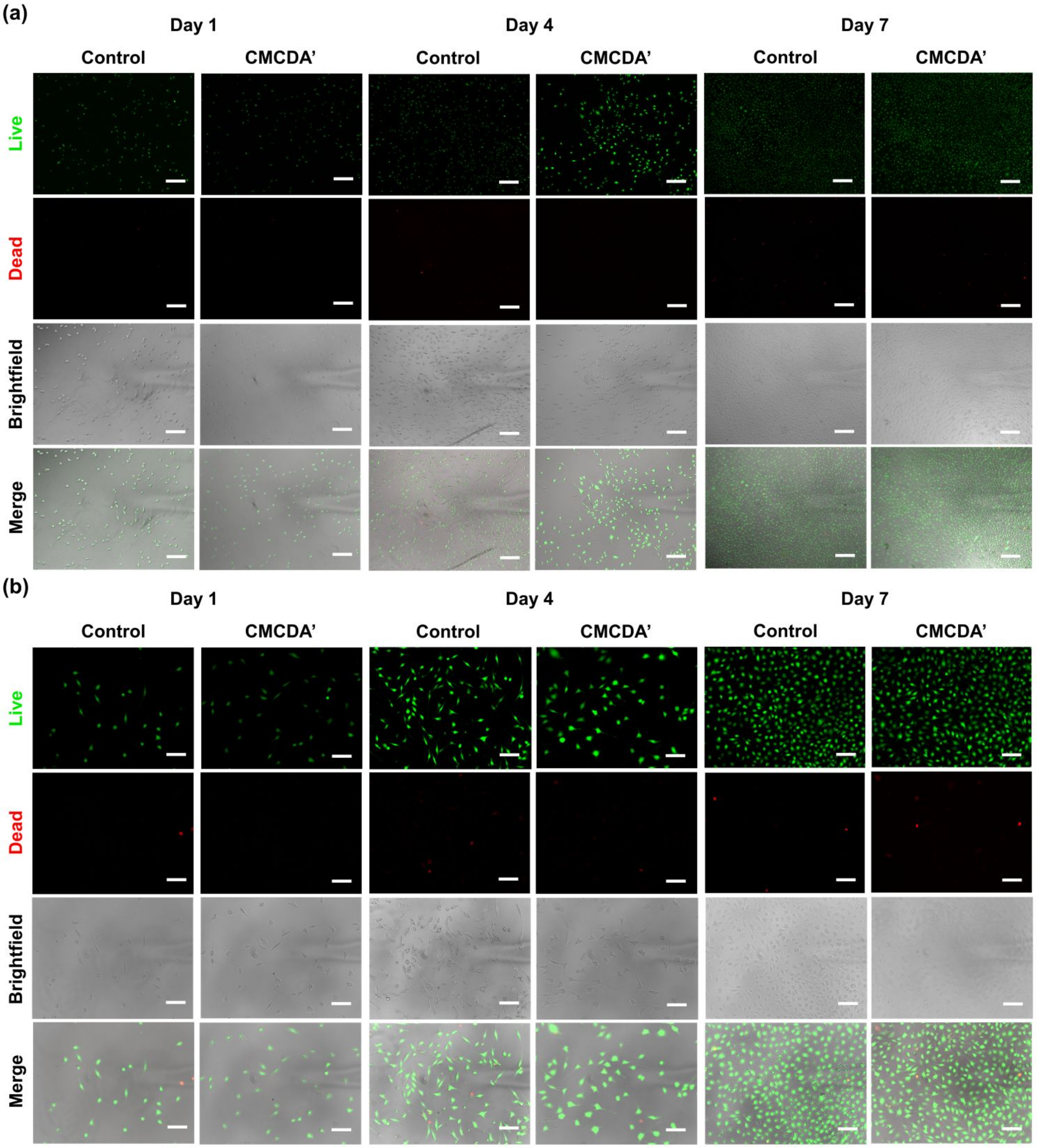

Figure S11. Direct contact test, morphological observation of L929 cells, and longer-term viability assessment. Live/dead staining images of L929 cells after 1, 4 and 7 days of exposure to the hydrogels, acquired at (a) 4X (scale bar: 200 μm) and 10X magnification (scale bar: 100 μm).

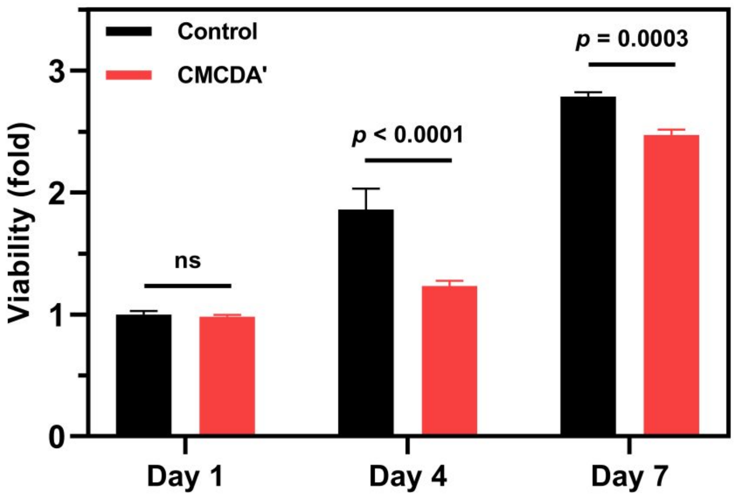

Figure S12. CCK-8 assay results for direct contact test of cell viability following 1, 4 and 7 days of exposure to the hydrogels. Data are shown as the mean values ± SD. Statistical significance was determined by two-way ANOVA with Tukey's post hoc test. *$p < 0.05$, **$p < 0.01$, ***$p < 0.001$, ****$p < 0.0001$; ns, not significant.
